# Designing a broad-spectrum multi-epitope subunit vaccine against leptospirosis using immunoinformatics and structural approaches

**DOI:** 10.3389/fimmu.2024.1503853

**Published:** 2025-01-28

**Authors:** Guneswar Sethi, Young Kyu Kim, Su-Cheol Han, Jeong Ho Hwang

**Affiliations:** ^1^ Animal Model Research Group, Korea Institute of Toxicology, Jeonguep, Jeollabuk-do, Republic of Korea; ^2^ Center for Companion Animal New Drug Development, Korea Institute of Toxicology, Jeonguep, Jeollabuk-do, Republic of Korea

**Keywords:** leptospirosis, multi-epitope subunit vaccine, immunoinformatics, population coverage, molecular docking, molecular dynamics simulation, *in silico* cloning

## Abstract

**Introduction:**

Leptospirosis, caused by *Leptospira interrogans*, is a neglected zoonotic disease that poses a significant global health risk to both humans and animals. The rise of antimicrobial resistance and the inefficacy of existing vaccines highlight the urgent need for new preventive strategies.

**Methods:**

An immunoinformatics approach was employed to design a multi-epitope subunit vaccine (MESV) against leptospirosis. B-cell, cytotoxic T lymphocyte (CTL), and helper T lymphocyte (HTL) epitopes were selected from five key *Leptospira* proteins. These epitopes were fused with a heparin-binding hemagglutinin (HBHA) adjuvant and appropriate linkers to construct the broad-spectrum vaccine. The physicochemical properties of the vaccine were assessed, including antigenicity, immunogenicity, allergenicity, and conservation. The vaccine’s 3D structure was modeled, optimized, and validated. Molecular docking, molecular dynamics simulations, and MM-GBSA analysis were performed to assess the vaccine's binding interactions with Toll-like receptors (TLR2 and TLR4). Immune simulations and *in silico* cloning were also conducted to evaluate the vaccine’s immune response and expression potential.

**Results:**

The MESV demonstrated high antigenicity, immunogenicity, non-allergenicity, and conservation across different *Leptospira* strains. Population coverage analysis revealed that T-cell epitopes significantly interacted with HLA molecules, covering 95.7% of the global population. Molecular docking showed strong and stable binding with TLR2 and TLR4, with binding energies of -1,357.1 kJ/mol and -1,163.7 kJ/mol, respectively. Molecular dynamics simulations and MM-GBSA analysis confirmed the stability of these interactions and accurately calculated the intermolecular binding free energies. Immune simulations indicated robust B and T cell responses, and *in silico* cloning demonstrated that the vaccine could be successfully expressed in *E. coli*.

**Discussion:**

These findings suggest that MESV is a promising candidate for leptospirosis prevention, providing robust immune responses and broad population coverage. However, further *in vivo* studies are necessary to validate its efficacy and safety.

## 1 Introduction

Leptospirosis is a widespread and reemerging zoonotic disease caused by spirochetes of the genus *Leptospira* (1). It affects an estimated 1 million individuals annually, resulting in approximately 60,000 fatalities worldwide (2, 3). The disease is particularly prevalent in tropical and subtropical regions, where environmental and socioeconomic factors such as poor sanitation, frequent flooding, and close human-animal interactions create ideal conditions for transmission (3). The disease manifests as flu-like symptoms or severe complications like Weil’s disease, leading to multi-organ failure (4, 5). Similarly, the disease has a significant economic impact on agriculture and companion animals, particularly in underdeveloped countries, where it can cause abortions, infertility, decreased milk production, and cattle death (6). Despite identifying 66 *Leptospira* species and over 300 pathogenic serovars (7, 8) grouped into 26 serogroups, effective treatment and prevention options remain limited (9). Currently, inactivated bacterial vaccines are the only approved prophylactic measure, but they offer limited cross-protection against diverse serovars and provide only short-term immunity (10, 11). Moreover, antibiotics such as azithromycin, doxycycline, penicillin, and cephalosporins are used to treat *Leptospira* infections. Still, challenges like antibiotic resistance and delayed diagnosis due to non-specific symptoms complicate disease management (12). This underscores the urgent need for a universal vaccination strategy.

Vaccination efforts have shown promise in countries like Cuba (13), Russia (14), and China (15). In Cuba, the Vax-Spiral^®^ vaccine, registered in 1998, became part of the National Leptospirosis Prevention and Control Program (16). A phase III clinical trial demonstrated 78.1% efficacy with no serious adverse effects (16, 17). However, these vaccines often fail to address the extensive diversity of *Leptospira* strains, and single-antigen formulations require frequent boosters, which can lead to side effects and leave gaps in widespread protection. Challenges also include local serovar variations, potential autoimmune reactions (e.g., uveitis) (18), and an incomplete understanding of protective immunity mechanisms. Moreover, *Leptospira*’s genetic diversity and immune evasion strategies, such as antigenic variation, complement evasion, and rapid tissue infiltration, make developing a broadly effective vaccine particularly complex. A key limitation of current approaches is the lack of a standardized animal model to evaluate human vaccine candidates, coupled with variability in immune responses across different populations and regions. Despite ongoing efforts, including clinical trials of multi-serovar and subunit vaccines, no universally protective vaccine has been established. These gaps underscore the need for innovative strategies to elicit sustained, cross-protective immunity against leptospirosis, particularly through the development of vaccines that can overcome antigenic diversity and immune evasion mechanisms.

Our study aims to address these gaps by designing a multi-epitope subunit vaccine (MESV) incorporating immunogenic proteins to provide broad-spectrum protection. Multi-epitope vaccine designs are gaining recognition for their potential to induce comprehensive immune responses while minimizing side effects against Leptospirosis (19–22). Previous studies, such as Majid et al. (19), demonstrated computationally designed MESVs’ potential to enhance immune responses through IFN-gamma induction. Additionally, Pankaj et al. explored proteins like LigA and LigB, which are promising candidates due to their roles in *Leptospira* virulence (20, 22).

Outer membrane proteins (OMPs) are promising vaccine candidates due to their surface exposure and involvement in virulence (23, 24). Notable OMPs, such as LipL32, LigA, and LigB, have demonstrated substantial protective efficacy in mouse models (17, 25). In this study, we selected five immunogenic proteins: LipL71, TonB-dependent receptor (TBDR), putative lipoprotein (irpA), sphingomyelinase C2 (Sph2), and general secretory pathway protein D (GspD) (26) as vaccine targets based on their roles in leptospiral pathogenesis and immunogenicity. LipL71 plays a role in pathogenesis by binding peptidoglycan and inducing antibody responses (27–29). TBDR and irpA are essential for iron uptake, survival, and virulence (30, 31). Sph2, a key virulence factor, causes host cell apoptosis and inflammatory tissue damage and serves as a diagnostic marker due to its early presence in infections (32, 33). GspD, a type 2 secretion system secretin, elicits bactericidal antibody responses, targeting multiple *Leptospira* species (34).

Advances in immunoinformatics enable rapid and cost-effective vaccine design, predicting immunogenic epitopes for robust immune responses (35). This approach has been successfully applied to pathogens like *Mycobacterium tuberculosis* (36, 37), *Plasmodium falciparu*m (38), and *Pseudomonas aeruginosa* (39). By integrating epitope prediction, TLR docking, and immune simulations, our study aims to overcome traditional vaccine development limitations and provide sustained, cross-protective immunity against leptospirosis.

## 2 Material and methods

### 2.1 Protein selection and retrieval

**Figure 1:**
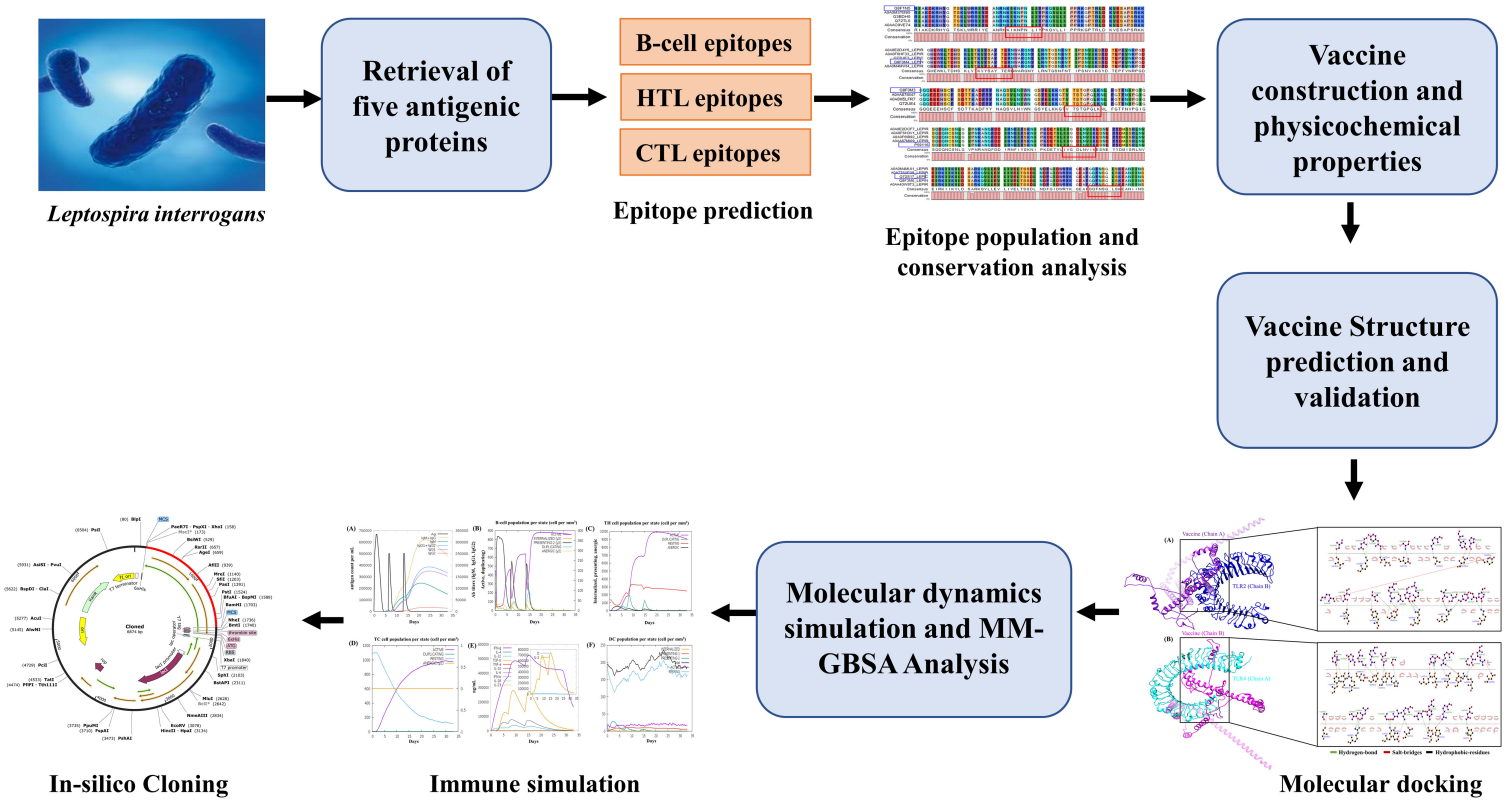
Methodology adopted for developing a multi-epitope subunit vaccine against *Leptospira interrogans*.

The prioritized target proteins, including LipL71 (UniProt ID: Q8F1N5), TBDR (UniProt ID: Q8F0M4), irpA (UniProt ID: Q8F0M3), Sph2 (UniProt ID: P59116), and GspD (UniProt ID: Q72S17), were retrieved from the UniProt database in FASTA format (40). For the vaccine design, we used a pathogenic strain of *L. interrogans* serogroup Icterohaemorrhagiae serovar Lai (strain 56601; Proteome ID: UP000001408). Subsequently, VaxiJen v2.0, an online prediction server, was used to identify the most potent antigenic protein through alignment-independent prediction with a threshold of 0.4 (41). Determining the subcellular localization and allergenicity of the selected proteins is essential for designing potential vaccine candidates. CELLO v.2.5 and PSORTb v3.0.3 were selected for their high accuracy for bacterial subcellular localization predictions, and allergenicity was evaluated using AllergenFP v.1.0 (42–44). To reduce the risk of autoimmunity, antigenic proteins were analyzed against the human proteome using the BLASTp tool with default parameters (45). Signal peptides were removed from the candidate proteins before the epitopes were predicted. We used the SignalP 5.0 server to identify and exclude these signal peptides. The workflow used in this study is presented in **Figure 1**. A detailed list of the databases, software, and web services utilized in this study is provided in **Supplementary Table S1**.

### 2.2 Prediction and screening of epitopes

To predict B-cell epitopes, we utilized the ABCpred server v.2.0, a bioinformatics tool well-regarded for predicting antigenic epitopes or antibody-binding sites within protein sequences (46). This tool was specifically chosen for its demonstrated reliability and high predictive accuracy across various pathogens, including bacteria, viruses, and parasites, making it suitable for vaccine candidate development. Using artificial neural networks (ANN), ABCPred predicts linear and continuous B-cell epitopes with high sensitivity and specificity. In our analysis, we applied a threshold of 0.85, generating 16-mer epitopes and focusing on top-scoring immunogenic epitopes for further investigation.

To predict HTL epitopes, which are crucial for immune system activation, we used the IEDB MHC II binding prediction server with the NetMHCIIpan 4.1 EL method (47, 48). This server is considered one of the most accurate tools for predicting peptide-MHC class II interactions based on a comprehensive set of experimentally validated epitopes. All parameters were kept at their default settings except for allele selection. We selected a reference set of HLA alleles covering 99% of the global allele distribution to ensure that the prediction results are applicable across diverse populations. Peptides with the lowest percentile ranks, indicating the highest predicted affinities, were then selected for further investigation. The IFNepitope and IL4Pred servers were used to predict Interferon-gamma (IFN-γ) and Interleukin-4 (IL-4) induction (49, 50), both of which are critical for assessing immune activation. These servers employ a combination of support vector machine (SVM) and motif-based approaches to differentiate between IFN-γ-producing and non-producing peptides. This step helped prioritize HTL epitopes most likely to trigger a strong immune response, which is crucial for vaccine efficacy.

NetCTL v1.2, with parameters for epitope identification (0.75), C-terminal cleavage (0.15), and TAP transport efficiency (0.05), was selected due to its integration of multiple prediction metrics for MHC class I binding (51). A3 and B44 supertypes were used for screening, ensuring high predictive coverage for CTL epitopes. Subsequently, we used the online tools VaxiJen 2.0, AllergenFP v.1.0, and ToxinPred (52) to evaluate the antigenicity, allergenicity, and toxicity of the selected B-cell, HTL, and CTL epitopes. Furthermore, the predicted epitopes were also cross-checked with the IEDB to identify known experimentally validated epitopes for *L. interrogans* (ID-173) (53, 54).

### 2.3 Epitope population and conservation analysis

The vaccine demographic reach of the CTL and HTL epitopes was evaluated using the IEDB population coverage analysis tool with default parameters (55). This tool can assess epitopes against their respective HLA genotype frequencies to ensure adequate coverage across the global human population. In designing the MESV, we evaluated the conservation of B-cell, HTL, and CTL epitopes across multiple *L. interrogans* strains. Using CLC Sequence Viewer v.8.0, we aligned the sequences of five selected proteins from various pathogenic strains with default parameters. WebLogo v.3 generated sequence logos highlighting conserved residues within the epitopes to visualize amino acid conservation and sequence preferences (56). The tool was used with default settings and a sequence weight of 1. Peptides were considered conserved if their amino acids consistently appeared across all analyzed strains. These conserved peptides were incorporated into the MESV to ensure broad applicability and effectiveness.

### 2.4 MESV construction and physicochemical property determination

Epitopes demonstrating high antigenicity, conservation, and non-toxicity were carefully selected for inclusion in the final MESV. Optimal B-cell, CTL, and HTL epitopes were selected based on superior scores and linked using appropriate linkers. To improve vaccine effectiveness, the heparin-binding hemagglutinin (HBHA; P9WIP9) sequence was obtained from the UniProt database and incorporated into the N-terminus as an adjuvant, using the EAAAK linker for facilitation. The KK and GPGPG linkers connected the B-cell and HTL epitopes, while CTL epitopes were linked using AAY linkers.

The physicochemical properties of the MESV, including isoelectric point (pI), molecular weight, and aliphatic index, were analyzed using the ProtParam tool (57). The ANTIGENpro server (58) was used for antigenicity prediction due to its pathogen-independent, sequence-agnostic approach, and the VaxiJen v2.0 server (41) provided an additional antigenicity assessment. AllerTOP v2.0, based on auto cross-covariance (ACC) transformation, was utilized to predict the allergenicity of the MESV sequence (59). Solubility predictions were made using SOLpro, an SVM-based tool, and Protein-Sol, which compares solubility profiles of input sequences against a solubility database (58, 60).

### 2.5 Structure modeling and validation

For vaccine development, understanding the 2D and 3D structures of the proposed MESV is essential, as these structures reveal functional characteristics and aid in docking studies. The 2D structure was modeled using PSIPRED v.4.0 and GORIV (61, 62). PSIPRED employs position-specific scoring matrices for precise sequence homology identification, while GORIV utilizes information theory and Bayesian statistics to provide complementary insights. For 3D structure prediction, trRosetta, integrating deep learning with the Rosetta platform, was selected due to its high precision in complex protein modeling (63). The modeled structure was further refined using the GalaxyRefine webserver. The server employs the ab initio method to model the missing loops and terminal ends (64). Model validation was conducted using ProSA-web for Z-score validation (65) and Ramachandran plot analysis with PROCHECK to assess stereochemical properties (66). The final 3D model was visualized with Chimera 1.17.1 to examine structural details (67).

### 2.6 Linear and conformational antibody epitope prediction

After creating the 3D model of the vaccine construct, continuous and discontinuous epitopes were predicted using the IEDB ElliPro online tool with default settings and an epitope threshold score of 0.5 (68).

### 2.7 Molecular docking

Toll-like receptors (TLRs), particularly TLR2 and TLR4, play a crucial role in recognizing *Leptospira* components and initiating immune responses (69). TLR4 specifically recognizes pathogen-associated molecular patterns (PAMPs), such as lipopolysaccharides, to trigger high-sensitivity immune responses (70). To investigate potential interactions between the MESV and TLRs, the crystal structures of TLR2 (PDB ID: 5D3I) and TLR4 (PDB ID: 4G8A) were retrieved from the Protein Data Bank. Pre-docking preparations were performed to remove non-essential molecules like water, minimizing interference during the binding assessment. The Dock Prep tool in UCSF Chimera v.1.17.1 refined the structures by adding hydrogen atoms and assigning proper atomic charges. For TLR4, the monomeric form was chosen to simplify the docking process with the vaccine construct. Docking was carried out using ClusPro 2.0 (71), from which the most favorable configuration based on binding energy was selected for further analysis. Visualization of receptor-vaccine interactions was carried out with UCSF Chimera 1.17.1 (64) and LigPlot+ v.2.2.5 (69), which was used to create 2D interaction maps displaying hydrophobic and hydrogen bonds between the vaccine and TLRs.

### 2.8 Molecular dynamics simulation

MDS was conducted using the GROMACS v2022 software package to examine the structural and binding stabilities of the MESV-TLR2 and MESV-TLR4 complexes. To perform the 100 ns simulation, we solvated the system with the GROMAS96 43a1 force field within a cubic box with 10.0 Å dimensions (72). The system’s charge was neutralized by adding Na^+^ and Cl^−^ ions. Energy minimization was performed using the steepest descent method and equilibrated through a 200 ps simulation at 300 K and 1 bar pressure in the NVT and NPT ensembles to remove unfavorable steric clashes. Van der Waals and electrostatic interactions were handled using the particle-mesh Ewald (PME) method with a 1 nm cutoff radius (73). LINCS and SETTLE algorithms were used to constrain the bond lengths and water geometry (74, 75). Temperature was controlled with the Berendsen thermostat using a V-rescale, and pressure was regulated using the Parrinello-Rahman method (76). The stability of the designed MESV was assessed using GROMACS tools by analyzing the root mean square deviation (RMSD), radius of gyration (Rg), root mean square fluctuation (RMSF), and solvent-accessible surface area (SASA). The binding energy of the MESV-TLR2 and MESV-TLR4 complexes was evaluated through the MM-GBSA method using the HawkDock server (77).

### 2.9 Immune simulation

*In silico* immune simulations were conducted using the C-ImmSim online server to assess the immune response characteristics of the vaccine design (78). These simulations generated specific immune responses to antigens using agent-based approaches, including positional score matrix and machine learning methods. Apart from time steps 1, 84, and 170, the simulation adhered to default parameters. Following the customary dosing intervals prevalent in conventional vaccine administration, three injections were administered at four-week intervals. This scheduling aligns with the optimal immune response induction guidelines and vaccine effectiveness. Plot analysis was conducted to determine the Simpson index (D), which served as a metric for diversity.

### 2.10 *In silico* cloning

Codon adaptation tools were used to address the differences in codon usage between humans and *E. coli* to enhance expression rates in the host system. Adjusting codon usage to align with that of prokaryotic organisms is essential for efficient expression (79). The sequence was optimized using the VectorBuilder Codon optimization tool to align the codon usage with *E. coli* strain K12 as the host. An ideal codon adaptation index (CAI) score and a GC content ranging from 30-70% were considered for this assessment. *XhoI* and *BamHI* restriction sites were added to the 5′ and 3′ ends of the vaccine sequence. The vaccine constructs were then cloned into the pET-28a (+) expression vector using SnapGene software, followed by a simulation of agarose gel electrophoresis.

## 3 Results

### 3.1 Protein selection

Five proteins from *L. interrogans* were selected based on a comprehensive literature review, and their corresponding FASTA sequences were retrieved. The selected proteins were subjected to antigenicity evaluation and subcellular localization prediction to gain insights into their immunogenic properties. Each prioritized protein displayed an antigenic score >0.4 and was predicted to localize to the outer membrane. The non-homology analysis confirmed that none of the selected proteins shared similarities with the host proteome. Additionally, the AllergenFP v.1.0 server analysis indicated that none of the selected proteins exhibited allergenic properties (**Supplementary Table S2**).

### 3.2 Prediction of immunogenic epitopes

Before epitope prediction, signal peptides were identified and removed from the candidate proteins (**Supplementary Figure S1**). B-cell epitope prediction is crucial in vaccine design, as these surface-exposed epitopes are essential for initiating antibody production and immune responses. The ABCpred server was used to identify 77 B-cell epitopes from five candidate proteins (Supplementary File 1). The top five epitopes were selected based on their high score, antigenicity, non-allergenicity, and non-toxicity predictions (**Table 1**).

**Table 1:** B-cell epitopes prediction for the input *Leptospira interrogans* protein sequences *via* ABCpred server.

Si. No	Protein name	Sequence	Start position	Score	Antigenicity	Allergenicity	Toxicity
**1**	LipL71	KIAGRDTKTEGNKNTK	367	0.90	2.316	Non-allergen	Non-toxic
**2**	TBDR	YSLAKSGNVRDHEVNN	440	0.94	1.223	Non-allergen	Non-toxic
**3**	irpA	FQATAARDTFCINLSE	360	0.93	1.095	Non-allergen	Non-toxic
**4**	Sph2	PRYVGVPFTWDAKTNE	363	0.96	0.809	Non-allergen	Non-toxic
**5**	GspD	NPVIQSEDLGSERKPP	252	0.95	0.660	Non-allergen	Non-toxic

Recognizing HTL epitopes is essential for creating an immunotherapeutic vaccine, given their pivotal role in triggering humoral and cell-mediated immune responses. HTL epitope screening was conducted by selecting epitopes with the lowest percentile rank and IC50 values using the reference set of MHC-II HLA alleles provided in **Supplementary Table S3**. This study identified the top five epitopes from the selected proteins, revealing percentile rank and IC_50_ values ranging from 0.01 to 0.36 and 5 to 49, respectively. Furthermore, the HTL epitopes exhibiting a positive score for IFN-γ and IL-4 prediction were selected (**Table 2**).

**Table 2:** Selected HTL epitopes from *L. interrogans* proteins, with predictions for toxicity, antigenicity, allergenicity, and stimulation of IFN-γ and IL-4 production.

Si. No	Allele	Epitope	Percentile rank	Antigenicity	Allergenicity	Toxicity	IFN-γ inducer	IL-4 inducer
**1**	HLA-DRB1*09:01	AEENLKAAEESRVAA	0.01	1.0740	Non-allergen	Non-toxic	Positive	Positive
**2**	HLA-DRB5*01:01	RSYRFVGAESRYQQD	0.03	0.5564	Non-allergen	Non-toxic	Positive	Positive
**3**	HLA-DRB3*02:02	AVTAFAANNNPTAAD	0.01	0.4767	Non-allergen	Non-toxic	Positive	Positive
**4**	HLA-DRB1*04:01	GKKFHVIGTHAQSQD	0.03	1.2025	Non-allergen	Non-toxic	Positive	Positive
**5**	HLA-DQA1*01:02/DQB1*06:02	LTVDNQEAEISVGQD	0.01	1.1496	Non-allergen	Non-toxic	Positive	Positive

Subsequently, we predicted CTL epitopes from the selected protein sequences using the NetCTL 1.2 server to assess the role of CTLs in immune activation. To enhance accuracy, residues in the signal peptide regions were excluded for the TBDR epitope sequence ‘SEETNKPIL’ and Sph2 sequence ‘YLLLFSLIR.’ This analysis identified the top 10 epitopes (**Table 3**). We focused on the HLA supertypes -B44 and -A3, as they can enhance immune responses and provide broad population coverage through effective HLA targeting [68,69]. To assess the number of experimentally validated epitopes for *L. interrogans*, we consulted the IEDB, which lists 46 known epitopes for this pathogen. Among these, two B-cell epitopes for Sphingomyelinase C2 (Sph2) were previously reported. However, upon cross-checking our computationally predicted B-cell epitopes for Sph2, we found none matched the reported ones, indicating that our identified B-cell epitope is novel. Furthermore, the B-cell, HTL, and CTL epitopes predicted for all five candidate proteins were not reported as known epitopes in the IEDB for *L. interrogans*, further highlighting their novelty. The detailed findings from the epitope screening are provided in Supplementary File 1.

**Table 3:** Prediction of CTL epitopes from input *L. interrogans* protein sequences using NetCTL-1.2, alongside antigenicity, allergenicity, and toxicity assessments.

Protein name	Epitope	HLA class I supertypes	Antigenicity	Allergenicity	Toxicity
LipL71	KIKNPNLIY	A3	1.03	Non-allergen	Non-toxic
	GEEENPENL	B44	0.88	Non-allergen	Non-toxic
TBDR	KVYSAYTER	A3	0.57	Non-allergen	Non-toxic
	HEVNNTKSL	B44	0.45	Non-allergen	Non-toxic
irpA	VTSTGPGLK	A3	1.62	Non-allergen	Non-toxic
	AQMTYANVL	B44	0.60	Non-allergen	Non-toxic
Sph2	IVGDLNVIK	A3	0.81	Non-allergen	Non-toxic
	IEEKIQYIF	B44	0.47	Non-allergen	Non-toxic
GspD	GQFNSGLSK	A3	0.64	Non-allergen	Non-toxic
	REIKTSISI	B44	0.77	Non-allergen	Non-toxic

### 3.3 Population and conservation analysis

**Figure 2:**
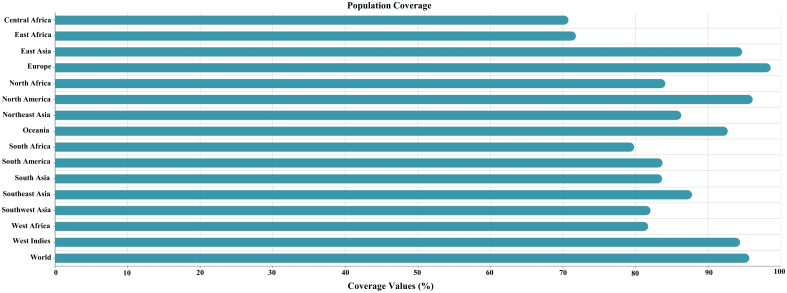
Global population coverage of the selected cytotoxic T lymphocyte (CTL) and helper T lymphocyte (HTL) epitopes. The x-axis represents the combined population coverage (HLA I and II), and the y-axis represents the countries.

The IEDB population coverage analysis tool was used to estimate the population coverage of the selected CTL and HTL epitopes. Globally, the combined epitopes had a population coverage of 95.7%. The highest coverage was found in Europe at 98.64%, and the lowest was in Central Africa, with a coverage of 70.78% (**Figure 2** and **Supplementary Table S4**).

**Figure 3:**
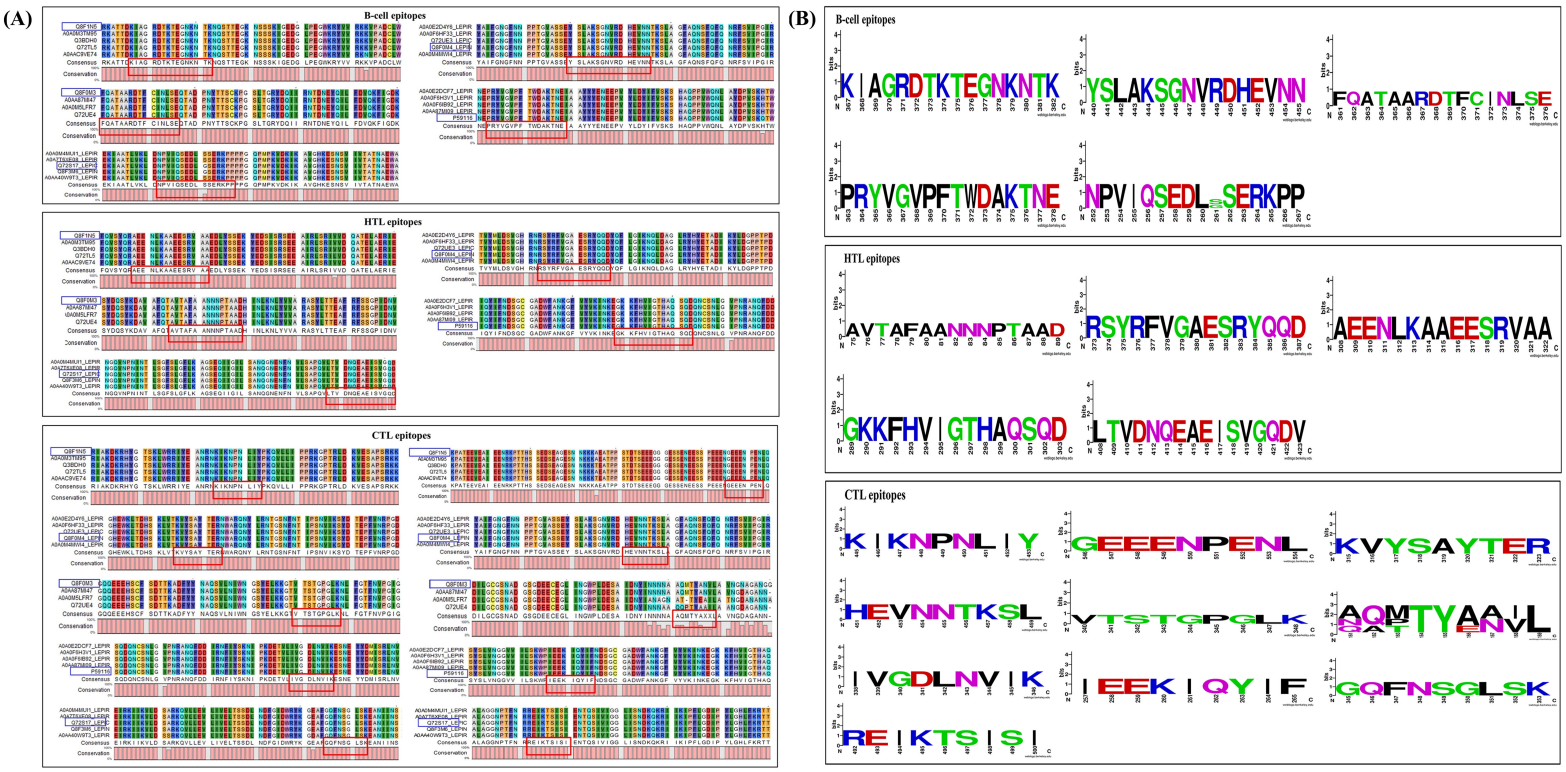
Epitope conservation analysis, featuring **(A)** alignments and consensus sequences for B-cell, HTL, and CTL epitopes associated with LipL71 (UniProt ID: Q8F1N5), TBDR (UniProt ID: Q8F0M4), irpA (UniProt ID: Q8F0M3), Sph2 (UniProt ID: P59116), and GspD, highlighted in red. The blue box indicates these epitopes, their corresponding proteins from other *L. interrogans strains*, and their UniProt IDs. **(B)** Sequence logo analysis of these epitopes for LipL71, TBDR, irpA, Sph2, and GspD. The x-axis represents the amino acid positions within the peptide sequences, while the y-axis indicates the frequency of each residue at a given position. The total height of the letters reflects the level of sequence conservation at each position.

We conducted a conservation analysis of the selected peptides to develop a universal multi-epitope antibacterial vaccine. Using the CLC Main Workbench, we analyzed the amino acid sequences of five selected proteins. B-cell, HTL, and CTL epitopes from the proteins Lipl71, TBDR, and Sph2 were 100% conserved across various strains. The B-cell and HTL epitopes of irpA were 100% conserved. Among the two CTL epitopes in irpA, CTL epitope 1 (VTSTGPGLK) was 100% conserved, whereas CTL epitope 2 (AQMTYANVL) was only 63.89% conserved. Similarly, for GspD, the HTL and CTL epitopes were 100% conserved. In contrast, the B-cell epitope was 97.5% conserved (**Figure 3A**). Additionally, we performed sequence logo analysis using WebLogo, which visually represents the frequency of each amino acid at specific positions across all sequences. This analysis revealed mutations in the CTL epitope ‘AQMTYANVL’ and B-cell epitope ‘NPVIQSEDLGSERKPP’. However, other epitopes showed high conservation (**Figure 3B**). These results highlight the potential of this vaccine for broad population coverage and its conserved efficacy across various pathogenic strains, indicating its global applicability and effectiveness.

### 3.4 Final vaccine construct and physiochemical properties

**Figure 4:**
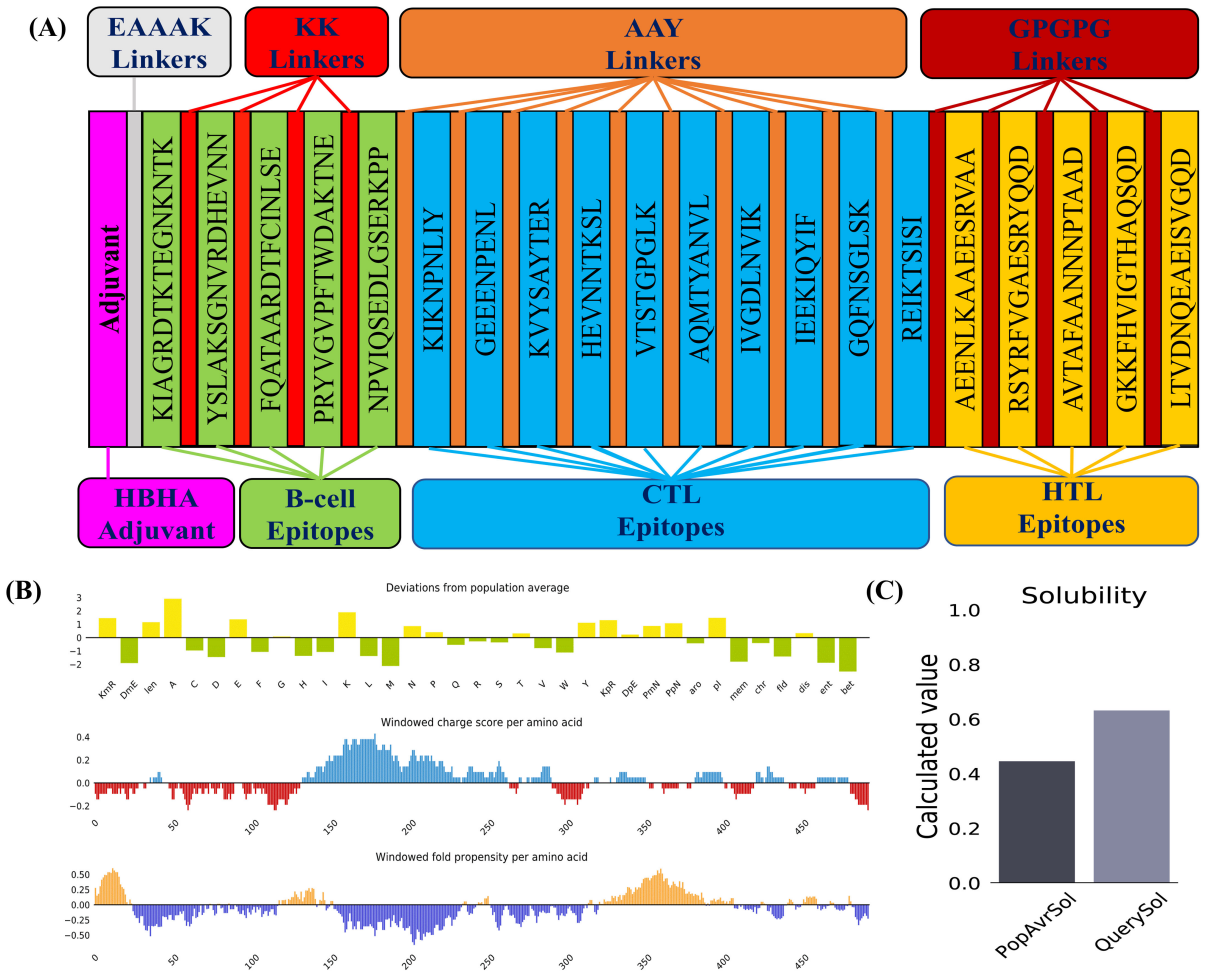
Vaccine construction and solubility prediction. **(A)** Diagrammatic representation of the critical components of the final vaccine construct. **(B)** Plot illustrating deviations from population averages across 35 features, including windowed charge scores and the folding propensity of amino acids. **(C)** Solubility prediction of the designed vaccine construct using the Protein-Sol server, compared to the population average across the analyzed datasets.

The MESV construct comprised one adjuvant (HBHA) with a length of 199 amino acids, five B-cell epitopes, five HTL epitopes, ten CTL epitopes, one EAAAK, four KK linkers, five AAY linkers, five GPGPG linkers. The resulting MESV had a total length of 589 amino acids (**Figure 4A**).

Physicochemical characterization of the MESV was evaluated using the ExPASy ProtParam tool. The instability index (34.04) and GRAVY score (-0.632) indicated that MESV was stable and hydrophilic, which are advantageous traits for a subunit vaccine. The proposed vaccine demonstrated notable antigenicity, with scores of 0.8409 (VaxiJen) and 0.929 (ANTIGENPro), confirming its potential as an antigen. The AllerTop server revealed that the designed construct was non-allergenic. Moreover, the vaccine attained solubility scores of 0.632 and 0.796, calculated using the Protein-Sol and SOLpro servers, respectively (**Table 4** and **Figures 4B, C**). Physicochemical evaluations suggested that the proposed vaccine exhibited favorable characteristics, making it a promising candidate for subsequent development.

**Table 4:** Physicochemical properties of the vaccine construct are predicted by the ExPASy Protparam tool.

Physiochemical Properties of Vaccine	Values	Comment
Number of amino acids	512	Appropriate
Molecular weight	55.69 kDa	Appropriate
Theoretical pI	9.08	Basic
Total number of negatively charged residues (Asp + Glu)	66	–
Total number of positively charged residues (Arg + Lys)	75	–
Total number of atoms	7755	–
Instability index	34.04	Stable
Aliphatic Index	72.09	Thermostable
Grand Average of Hydropathicity (GRAVY)	-0.632	Hydrophilic
Antigenicity (VaxiJen)	0.8409	Antigenic
Antigenicity (ANTIGENpro)	0.929	Antigenic
Allergenicity (AllerTOP)	Non-allergen	Non-allergenic
Solubility (Protein_Sol)	0.632	Soluble
Solubility (SOLPro)	0.796	Soluble

### 3.5 Vaccine structure analysis

**Figure 5:**
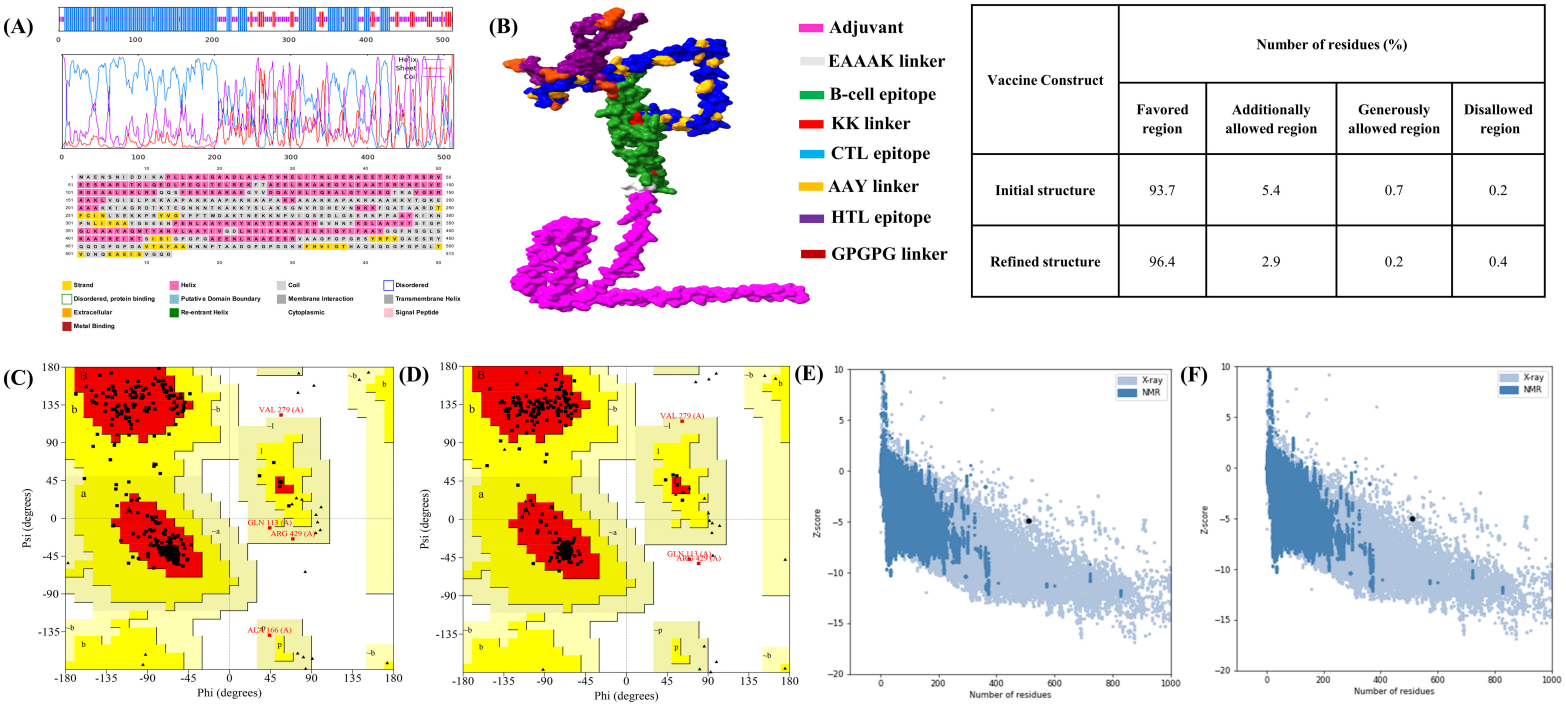
Predicted 2D and 3D structure of the vaccine construct. **(A)** Graphical representation of the secondary structure predicted using the PSIPRED server. GOR IV server results indicate that the vaccine consists of α-helix (57.50%), coil (30.99%), and β-strand (11.50%). **(B)** Predicted 3D structure of the designed multi-epitope subunit vaccine. **(C, D)** Ramachandran plots showing the distribution of amino acids in the favored, allowed, and disallowed regions before and after refinement. **(E, F)** Z-scores of the vaccine model before and after refinement.

The 2D structure of the designed vaccine construct was analyzed using the GOR IV and PSIPRED webservers. The findings indicate that MESV comprises 66.04% α-helix, 26.99% coil, and 6.96% β-strand (**Figure 5A**). The 3D structural coordinates of the MESV were generated using trRosetta and are shown in **Figure 5B**. Subsequently, the 3D structure quality was enhanced, as evidenced by the Ramachandran plots. **Figures 5C, D** show the validation results before and after refinement. The Ramachandran plot of the refined MESV models showed that 96.4% of the residues were located in the most favored regions. The ProSA server initially revealed a Z-score of -4.93 for the model, which improved to -5.03 following refinement (**Figures 5E, F**).

### 3.6 Determination of linear and conformational antibody epitopes

Determining linear and conformational (discontinuous) antibody epitopes is vital for understanding antigen-antibody interactions after developing a 3D vaccine model. The ElliPro server was used to predict epitopes in the 3D structure of the vaccine construct. Six continuous epitopes of various lengths were identified (**Supplementary Table S5** and **Figure S2A**) alongside seven discontinuous epitopes (**Supplementary Table S6** and **Figure S2B-H**).

### 3.7 Molecular docking and simulation study

**Figure 6:**
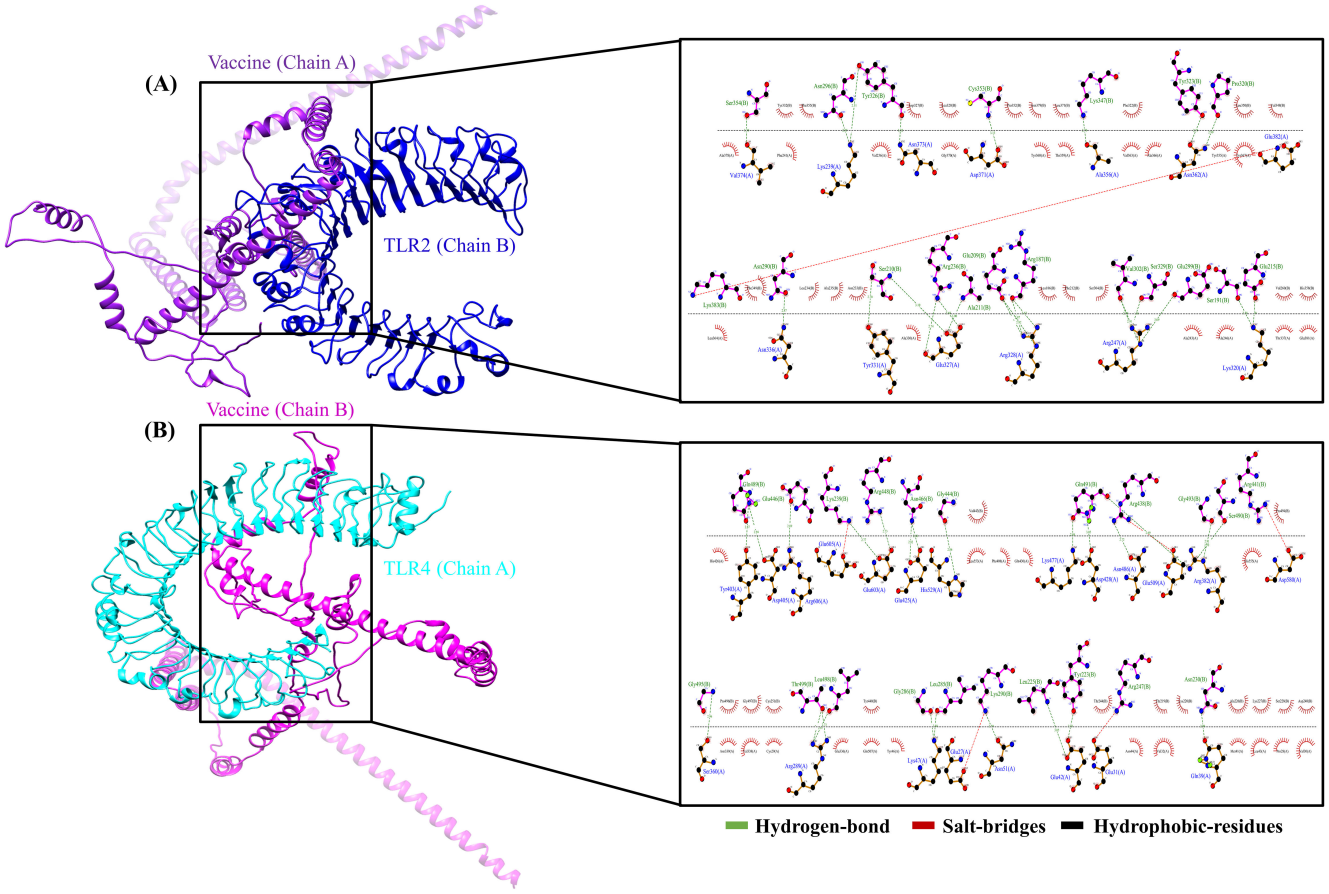
Molecular docking of the vaccine construct with immune receptors (Toll-like receptors (TLR) 2 and 4). **(A)** Cartoon representation of the vaccine-TLR2 complex, showing the interaction analysis between the vaccine (chain A) and TLR2 (chain B) using LigPlot. **(B)** The vaccine-TLR4 complex was illustrated using Chimera software, depicting the bond interactions between TLR4 (chain A) and the vaccine (chain B).

The interaction between MESV and immune receptors is crucial because vaccine components should bind to immunoreceptors to initiate protective immune responses by activating various immunomodulatory pathways. This interaction was accessed by molecular docking using ClusPro 2.0, with the lowest energy cluster considered optimal. TLR2 and TLR4 showed minimum energy values of -1,357.1 kJ/mol and -1,163.7 kJ/mol, respectively. LigPlot v.2.2.5 analysis further revealed specific interactions between MESV amino acids and TLR2 and TLR4 (**Figures 6A, B**). For the MESV-TLR2 complex, 23 hydrogen bonds and one salt bridge were identified. In contrast, the MESV-TLR4 complex formed 24 hydrogen bonds and five salt bridges (**Table 5**). These findings highlighted the potential effectiveness of the MESV vaccine in generating a targeted and strong adaptive immune response against leptospirosis.

**Table 5:** Molecular docking results and predicted hydrogen bond interactions and salt bridges between the vaccine and immune receptors.

Docked complex	Type of interaction	Interacting residue (Vaccine)	Interacting residue (TLR)
**Vaccine-TLR2**	Hydrogen bond	Lys239, Arg247, Lys320, Arg328, Glu327, Tyr331, Asn336, Ala356, Asn362, Asp371, Asn373, Val374, Glu382	Arg187, Ser191, Glu209, Ser210, Ala211, Glu215, Arg236, Asn290, Asn296, Glu299, Val302, Pro320, Tyr323, Tyr326, Ser329, Lys347, Cys353, Ser354
	Salt bridge	Glu382	Lys383
**Vaccine-TLR4**	Hydrogen bond	Tyr223, Leu225, Asn230, Lys239, Leu 285, Gly286, Lys290, Glu446, Arg448, Asn466, Gln489, Gly495, Leu498, Thr499,	Gln39, Glu42, Lys47, Asn51, Arg289, Ser360, Arg382, Tyr403, Asp405, Glu425, Asp428, Lys477, Asn486, His529, Arg606,
	Salt bridge	Lys239, Arg247, Lys290, Arg438, Arg441,	Glu27, Glu31, Glu509, Asp580, Glu605,

The structural stability of the MESV-TLR complexes was validated using MD simulations, which demonstrated the ability of the vaccine to bind to immune receptors and its potential to induce immunity over a 100 ns period. For the MESV-TLR2 complex, the backbone RMSD plot indicated minor deviations between 38 and 60 ns; however, the system stabilized after, maintaining an RMSD between 2.5 and 3.0 Å (**Figure 7A**). In contrast, the MESV-TLR4 complex demonstrated consistent stability throughout the simulation, with the backbone RMSD remaining steady at an average value of 2.50 Å (**Figure 7B**). RMSF analysis revealed that the TLR4 and vaccine backbone exhibited fewer fluctuations (**Figure 7D**). In contrast, the TLR2 backbone, particularly chain B, showed greater dynamics and fluctuations (**Figure 7C**). Additionally, the vaccine backbone exhibits increased dynamics when bound to TLR2. In contrast, it remained more stable during the interaction with TLR4. The average Rg for the MESV-TLR2 and MESV-TLR4 complexes was calculated as 4.4 and 4.2 nm, respectively. Rg analysis revealed that the TLR2 complex displayed slightly different behavior over the 100 ns simulation, whereas the TLR4 complex remained consistently folded throughout the process (**Supplementary Figure S3A, B**). The average SASA values for the MESV bound to TLR2 and TLR4 were determined to be 4.75 nm² and 5.10 nm², respectively (**Supplementary Figure S3C, D**). Furthermore, we analyzed the interaction between the vaccine and TLRs at intervals of 0, 20, 40, 60, and 100 ns. The results demonstrated that the immune receptors, TLR2 and TLR4, effectively bound to the vaccine (**Figure 8**).

**Figure 7:**
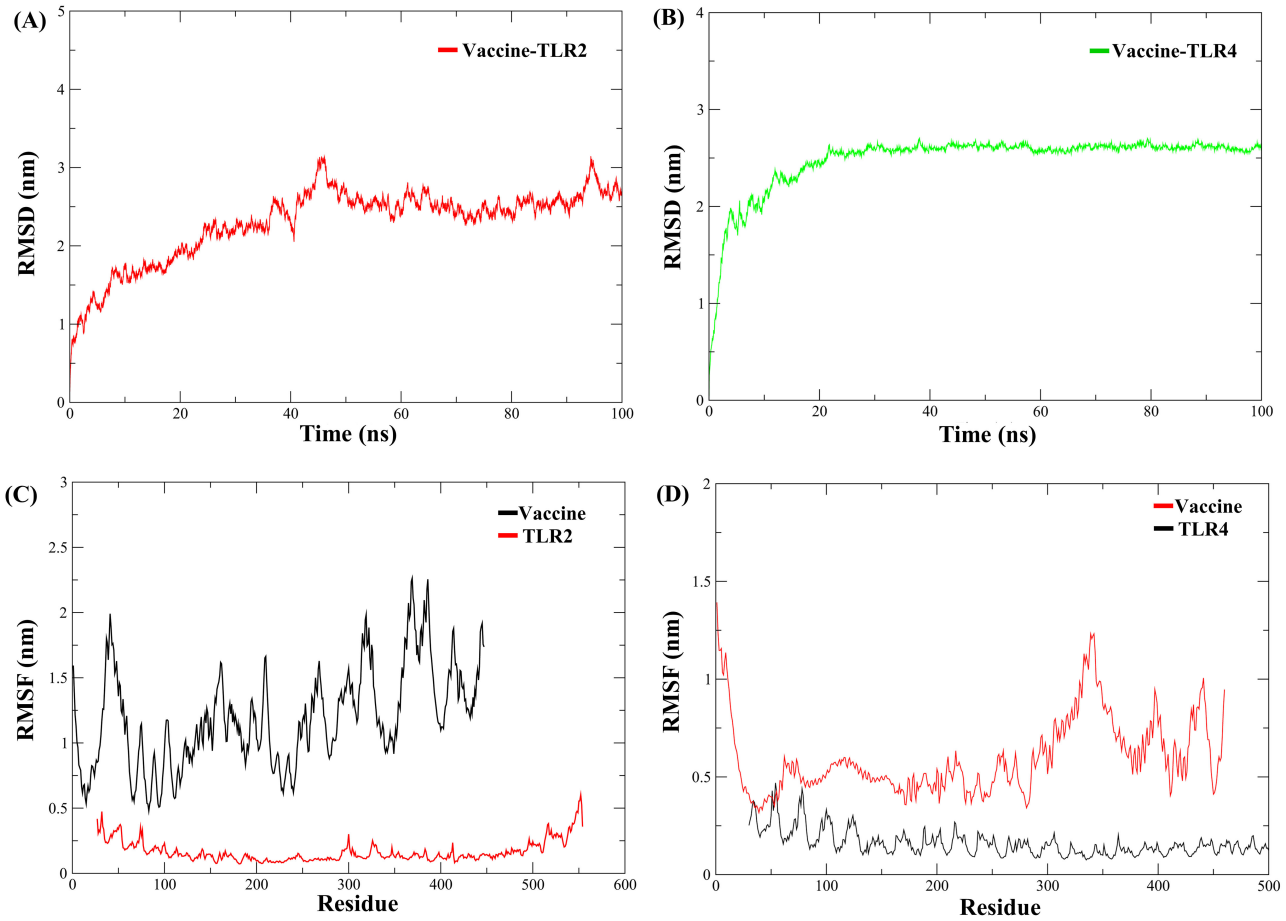
Molecular dynamics simulation of the vaccine complexed with immune receptors. **(A)** Root mean square deviation (RMSD) plot of the vaccine-TLR2 docked complex. **(B)** RMSD plot of the vaccine-TLR4 docked complex. **(C)** Root mean square fluctuation (RMSF) plot showing side-chain fluctuations within the vaccine-TLR2 complex. **(D)** RMSF plot illustrating side-chain fluctuations within the vaccine-TLR4 complex.

**Figure 8:**
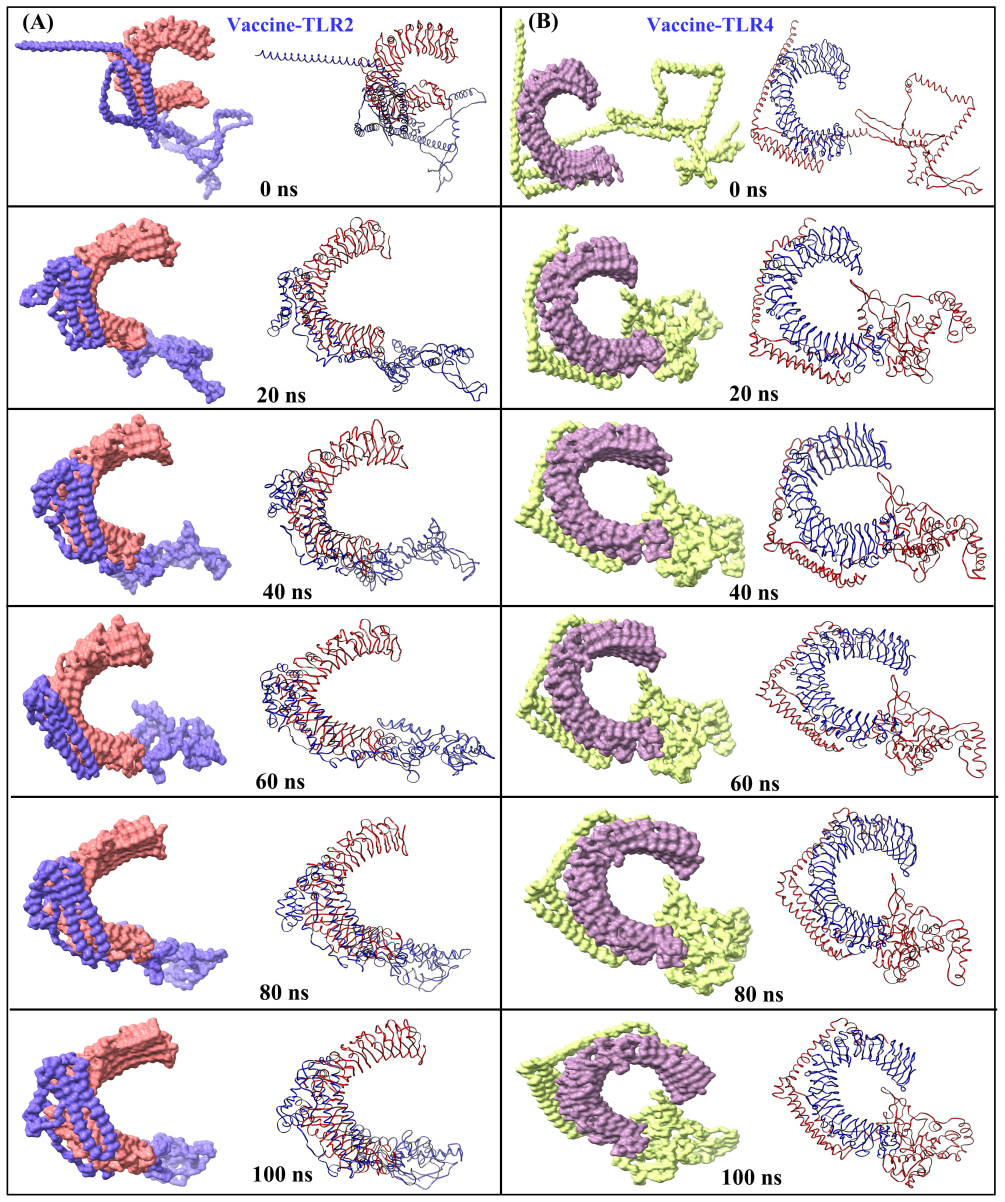
Snapshots of equilibrated (initial) systems and last trajectories: vaccine-bound complexes of **(A)** TLR2 and **(B)** TLR4 (surface and cartoons are shown for each snapshot).

In addition, the binding energy was calculated by incorporating the specific interactions between each residue. This assessment revealed substantial energy contributions from the interactions with MESV and TLR protein residues. The significantly more negative total binding energy indicated a higher affinity of the vaccine for the TLR4 interface (-152.63 kcal/mol) than with TLR2 (-122.92 kcal/mol) (**Table 6**). These findings suggest stable and favorable interactions within the vaccine-receptor complexes throughout the simulation.

**Table 6:** Binding free energies of the docked complexes were calculated *via* MM-GBSA analysis, and all energy values are provided in kcal/mol.

MM/GBSA calculations (Kcal/mol)		
Energy parameters	Vaccine-TLR2	Vaccine-TLR4
**VDW**	-194.42	-280.52
**ELE**	-2488.49	-3847.97
**GB**	2584	4012.7
**SA**	-24.84	-36.83
**Total binding energy**	-122.92	-152.63

*VDW, Van der Waals potential; *ELE, Electrostatic potential; *GB, Polar Solvation free energy predicted using Generalized Born model; *SA, The empirical model calculated the nonpolar contribution to the solvation free energy; *TOTAL, Final estimated binding free energy (kcal/mol).

### 3.8 Immune simulation

**Figure 9:**
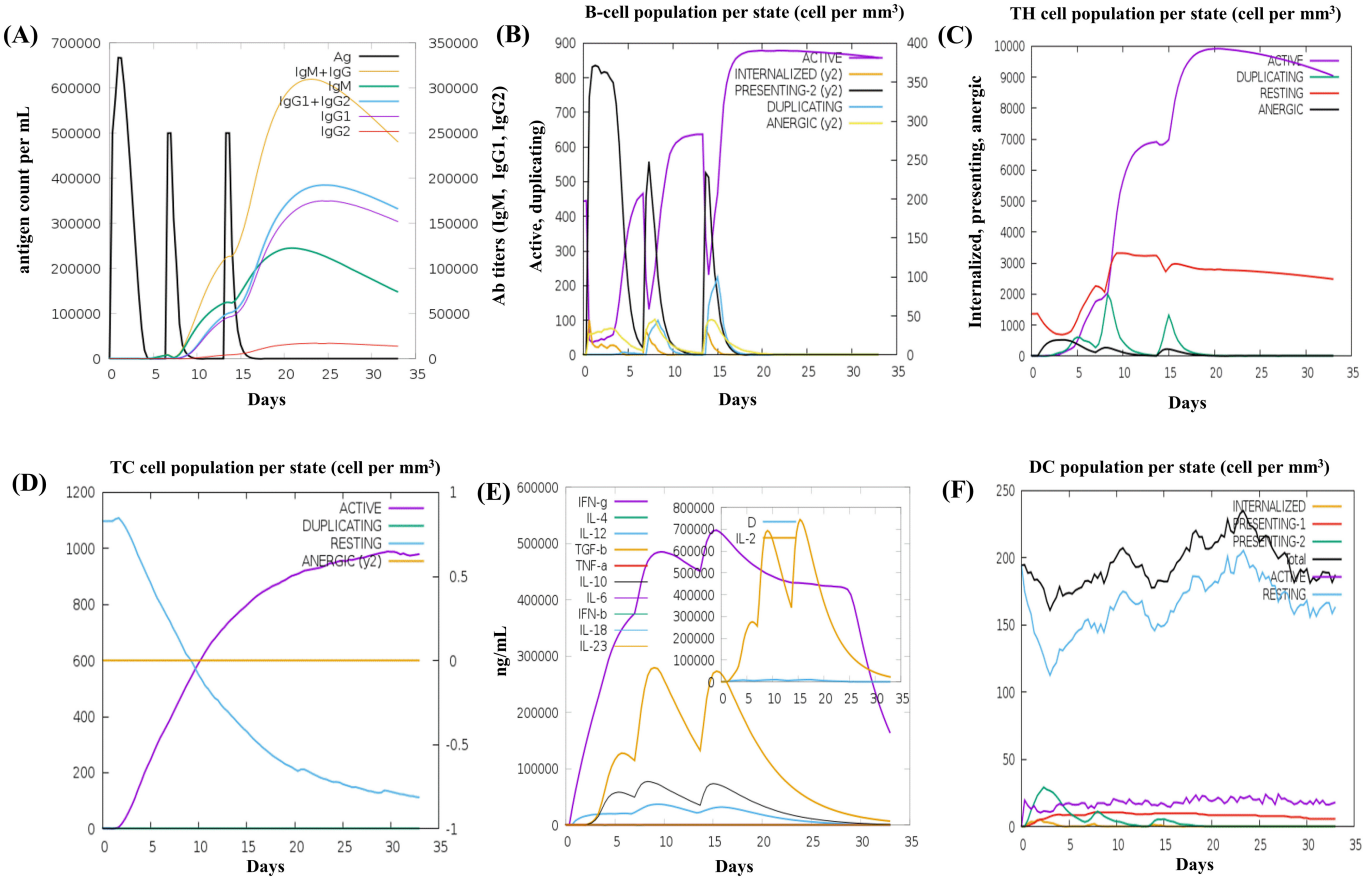
*In silico* immune simulation results of the vaccine construct using the C-ImmSim tool **(A)** The immunoglobulin response to antigen administration, depicted with colored peaks representing different immunoglobulin subclasses. **(B)** Active B-cell population observed after vaccination. **(C)** Generation of cytotoxic T cells in response to vaccination. **(D)** The emergence of helper T cells. **(E)** Graph showing the cytokine levels triggered by the vaccine, with an inset depicting the Simpson Index [D] for interleukin (IL)-2, used to measure diversity. **(F)** The dendritic cell population per state.

The C-ImmSim server was used to generate the immune response profile for MESV. Following the three vaccine doses, the antibody response significantly increased, with elevated IgM and IgG antibodies, indicating a strong humoral immune response. Higher IgG1, IgG2, and IgM expressions were associated with increased B-cell density, reduced antigen concentration, and a notable increase in memory B-cells (**Figures 9A, B**). Similarly, the data showed the development of secondary and tertiary immune responses, with an increase in the density of helper and cytotoxic T-cells (**Figures 9C, D**). These findings suggested a robust secondary immune response, enhanced antigen clearance, and effective immune memory formation following each dose. Moreover, during the dosing period, IFN-γ and IL-2 levels were elevated post-immunization (**Figure 9E**). After vaccination, the number of resting dendritic cells increased, and the antigen-presenting dendritic cells (types 1 and 2) decreased (**Figure 9F**). These results demonstrated that the vaccine design effectively elicited robust immune responses against leptospirosis.

### 3.9 Vaccine optimization and cloning

**Figure 10:**
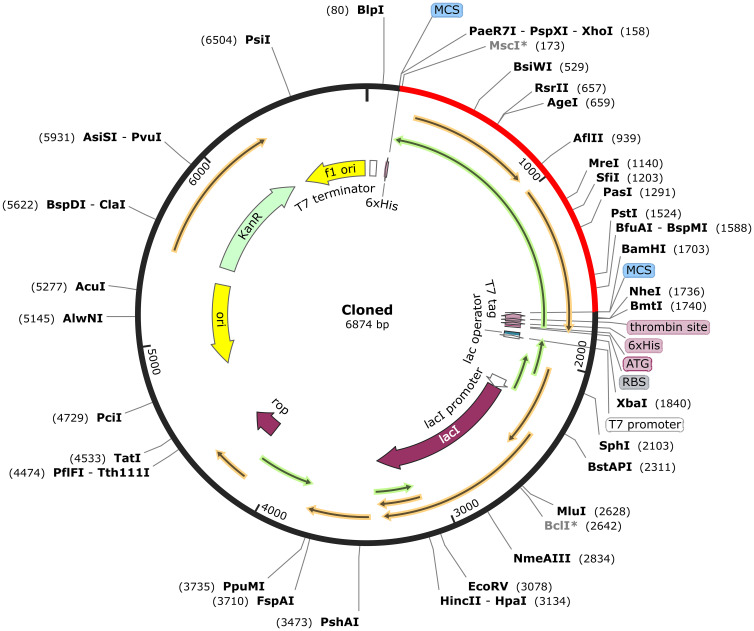
*In silico* cloning of the vaccine sequence into the pET-28a (+) expression vector. The vaccine sequence (red) was cloned between *XhoI* and *BamHI* restriction sites in the pET-28a (+) expression vector (black).

The MESV sequence was subjected to codon optimization to enhance its expression efficiency in the chosen expression system (*E. coli* K12). This optimization yielded a GC content of 51.83%, and the vaccine CAI was calculated as 0.954, implying a favorable expression outcome in the host organism. To facilitate cloning, *XhoI and BamHI* restriction sites were introduced at the beginning and end of the codon-optimized sequence, respectively. The cloning study involved the use of pET28a (+) plasmid. Our study used the pET-28a (+) vector due to its strong T7 promoter, facilitating high-level gene expression in *E. coli*. The vector’s His-6 tag simplifies protein purification, and the ampicillin resistance gene enhances selection efficiency. The MESV sequence, with a length of 6,874 bp, was constructed by integrating a 1,551 bp gene sequence. This process ensured efficient vaccine expression in the selected host system (**Figure 10**). Additionally, the size of the cloning product was verified using the simulated agarose gel feature of SnapGene software (**Supplementary Figure S4**).

## 4 Discussion

Leptospirosis, a globally significant zoonotic disease, poses a severe public health concern due to its diverse clinical presentations. The high fatality rate in severe cases, coupled with the lack of early diagnosis and effective treatment options, emphasizes the urgent need for novel preventive strategies. Current vaccines, primarily based on whole-cell inactivated leptospires, offer limited cross-protection among serovars and are associated with adverse effects. Advances in immunoinformatics and molecular biology have paved the way for multi-epitope subunit vaccines (MESV) that target conserved proteins across different strains. These innovative vaccines have the potential to overcome the shortcomings of traditional bacterin-based vaccines by providing broader protection, eliciting stronger, longer-lasting immune responses, and reducing adverse effects.

The development of effective vaccines for *Leptospira* is complicated by the pathogen’s genetic diversity and the limited number of experimentally validated epitopes. We consulted the Immune Epitope Database (IEDB) to address these challenges, which lists 46 known epitopes for *L. interrogans*. The IEDB has previously characterized two linear B-cell epitopes, Sph2 (176–191) and Sph2 (446–459), which have been shown to elicit immune responses in human leptospirosis (80). When we explored the IEDB, we found that all of our predicted B-cell, HTL, and CTL epitopes spanning multiple proteins, including Sph2, LipL71 (Q8F1N5), TBDR (Q8F0M4), irpA (Q8F0M3), and GspD (Q72S17) have not been reported before. This confirms that these epitopes are novel and distinct. Importantly, these newly identified epitopes show strong potential for inducing immune responses against *L. interrogans*, further supporting their suitability as vaccine targets. We also cross-checked our predicted epitopes for Sph2 with those previously reported and found no overlap with the known Sph2 (176–191) and Sph2 (446–459) epitopes. This comprehensive validation process ensures that our multi-epitope vaccine design is based on novel targets, which could enhance immunogenicity and broaden protection across different *Leptospira* serovars. Several studies have made notable contributions to *in-silico* vaccine design for leptospirosis. Lata et al. (21) explored the *Leptospira* proteome and identified lipoprotein Q75FL0 as a promising vaccine candidate. Similarly, Lin et al. developed a multi-epitope chimeric vaccine (r4R) using B and T-cell epitopes from LipL32, OmpL1, and LipL21, demonstrating its potential as a cross-reactive, protective antigen against leptospirosis (81). Abdullah et al. used an integrated vaccinomics approach to design a multi-epitope vaccine with B and T-cell epitopes, selecting six proteins (NP_712625, NP_714239, WP_011669637, WP_011670051, WP_011670465, WP_011671327), thus contributing additional vaccine candidates (82). Fernandes et al. (83) developed a chimeric multi-epitope protein (rChi) using five *Leptospira* proteins (LigA, Mce, Lsa45, OmpL1, and LipL41), demonstrating its potential to protect against lethal leptospirosis infection in a hamster model. Ibrahim et al. designed an Lsa46-based multi-epitope peptide vaccine against leptospirosis using an immunoinformatic approach (84). Our study extends these efforts by introducing novel epitopes from LipL71, TBDR, irpA, and GspD, proteins not previously explored in vaccine design. While studies by Majid et al. (19) and Pankaj et al. (22) focused on more widely studied proteins such as Hap1, LigA, LAg42, SphH, HSP58, and the LipL, LigA, and LigB family lipoproteins, our work broadens the pool of candidate antigens by identifying novel targets that have not been addressed previously. These newly identified proteins are involved in key *Leptospira* virulence mechanisms, including immune evasion and nutrient acquisition, making them strong candidates for vaccine development (26). In addition, our research incorporates population coverage analysis, providing a more comprehensive evaluation of the potential global applicability of the vaccine, which was not present in earlier studies.

In response to the urgent need for an effective leptospirosis vaccine, we designed an MESV incorporating five immunogenic antigens, predicting the B-cell, HTL, and CTL epitopes. Previous studies have demonstrated that γδ T cells from humans and bovines can proliferate and produce IFN-γ in response to *Leptospira* (85). Consistent with these findings, the identified epitopes in this study showed the potential to induce both IFN-γ and IL-4, as indicated by their positive scores. This highlights their capacity to trigger an IFN-γ-mediated immune response, reinforcing the vaccine’s ability to activate essential immune pathways against *Leptospira*. Each epitope was meticulously screened for allergenicity, toxicity, and antigenicity to ensure they could effectively elicit the desired immune response without causing adverse effects. Conservation analysis revealed that most B-cell, HTL, and CTL epitopes in the selected proteins were highly conserved across *Leptospira* strains, ensuring broad coverage. Minor mutations were observed in specific CTL and B-cell epitopes. The CTL and HTL epitopes were also selected according to the predicted HLA alleles specific to various ethnic groups. The results indicated that the selected epitopes provided a global population coverage of 99.77%, encompassing diverse geographic regions. Linkers such as AAY, GPGPG, and KK have been used to connect CTL, HTL, and B-cell binding epitopes in constructing the MESV. The adjuvant and vaccine sequences were joined using the EAAAK linker, which provided structural stiffness and maintained consistent spacing between them. Its α-helical structure reduces domain interference, encouraging proper folding and enhancing the fusion protein’s stability and functionality (86). Several studies have shown that adding GPGPG and AAY linkers between anticipated HTL and CTL epitope sequences induces junctional immunogenicity, allowing for the logical design and production of a potent MESV (36, 87). Previous studies have indicated that the KK linker prevents the development of antibodies against the amino acid sequence created by integrating two peptides. This enabled the antibody to recognize each peptide individually (88). Adjuvants, also known as innate immune stimulants, are selected based on their ability to elicit specific immunological responses. In our study, we chose HBHA due to its affinity for TLR4, which activates dendritic cells and skews the immune response towards a Th1-type profile (89)**. T**his is particularly advantageous for combating intracellular pathogens like *Leptospira interrogans*, which rely on strong cellular immunity for effective control (17). Moreover, HBHA has been shown to enhance the immunogenicity of peptide-based vaccines, making it an ideal candidate to amplify innate and adaptive immune responses (22). By incorporating HBHA, our multi-epitope vaccine strategy aims to elicit a more potent and targeted immune response against *Leptospira*.

MESV physicochemical analysis revealed promising characteristics with strong antigenic properties sufficient to trigger an effective immune response. Various server evaluations confirmed the vaccines’ high solubility and hydrophilicity. Additionally, the instability index of the vaccines was found to be within the acceptable range (below 40), indicating that they would remain stable within the host organism. Helices are essential for biomolecular recognition and play a key role in protein synthesis (90). In a previous MESV design against *L. interrogans* by Pankaj et al. (22), the presence of α-helix was reported to be 16.96%. In contrast, our designed vaccine model demonstrated a significantly higher α-helix content of 66.04%. This increased helix presence may enhance the stability and efficacy of the vaccine. The MESV’s 3D structure was predicted and refined using the GalaxyRefine server, resulting in significant quality improvements, with most residues positioned in the favored and allowed regions of the Ramachandran plot.

TLR2 and TLR4 receptors in vaccines boost protection against various pathogens by improving antigen presentation to T cells, causing the activation of CD4^+^ and CD8^+^ T cells, which are vital for a robust adaptive immune response. Additionally, existing literature emphasizes the crucial involvement of immune receptors, particularly TLR2 and TLR4, in coordinating host immunity against *Leptospira* infection. TLR2 mainly plays a predominant role in recognizing lipoproteins, highlighting its specific involvement in the immune response mechanisms against *Leptospira* (91, 92). Recent studies also show that TLR2 and TLR4 recognize *Leptospira* strains used in canine vaccine production, contributing to understanding innate immune responses in dogs, humans, and mice (69). Our observations showed that MESV interacts strongly with TLR2 and TLR4, with protein-protein docking revealing multiple hydrogen bonds and salt bridges. Notably, the vaccine exhibited higher binding energy with TLR4 than TLR2, indicating stronger interactions with TLR4. These findings highlight the potential effectiveness of the MESV in generating a targeted and strong adaptive immune response against leptospirosis.

The structural stability of the MESV-TLR complexes was validated using MD simulations, which demonstrated the vaccine’s ability to bind with immune receptors and its potential to induce immunity. The simulations showed that the MESV-TLR2 complex had a lower RMSD than the MESV-TLR4 complex, indicating high stability. The RMSF plot revealed that the regions involved in MESV-TLR interactions were less flexible. Moreover, the negative binding energies of MESV and receptors in the MMGBSA study reinforced the stability of the complexes. Immune simulation results indicated significant increases in B and Th-cell populations, with elevated levels of TGF-β, IL-10, and IFN-γ, essential for managing inflammation and controlling leptospirosis. Pankaj et al. (22) previously reported strong cellular and humoral responses, including elevated B-cell populations and IFN-γ levels. Our study expands on these findings by examining the role of T cytotoxic and dendritic cells, which demonstrated enhanced activation. This broader immune response highlights the vaccine’s potential for leptospirosis immunization. Induction of cytokines like IL-10 is vital for maintaining immune balance, while TGF-β helps regulate inflammation. The vaccine’s ability to increase B and Th-cell populations, alongside substantial production of TGF-β, IL-10, and IFN-γ, supports its efficacy in controlling disease progression. Furthermore, *in silico* cloning analysis indicated that MESV could be efficiently expressed in *E. coli* as a host system. However, further *in vivo* studies are needed to validate the efficacy and safety of this vaccine.

## 4 Conclusion

This study emphasizes the effectiveness of computational immunology in optimizing vaccine design. Our MESV against Leptospirosis demonstrated strong antigenicity, conservation, and safety, successfully eliciting robust immune responses, and showed broad population coverage with strong interactions with TLR4 and TLR2. Additionally, successful *in silico* cloning in *E. coli* supports its feasibility for expression. However, while the *in silico* approach yielded promising results, several limitations must be acknowledged. The antigenic variability among leptospirosis serovars and the reliance on computational predictions, which may not fully reflect complex biological interactions, emphasize the need for *in vivo* validation. Further research is required to address potential differences in vaccine effectiveness across diverse populations and challenges in expressing the vaccine in *E. coli*. These results underscore the importance of further experimental validation while demonstrating the valuable role of immunoinformatics in advancing vaccine development.

## Data Availability

The datasets presented in this study can be found in online repositories. The names of the repository/repositories and accession number(s) can be found in the article/**Supplementary Material**.
