# Supplementary material for: Designing a broad-spectrum multi-epitope subunit vaccine against leptospirosis using immunoinformatics and structural approaches
Source: Front Immunol. 2025 Jan 28;15:1503853. doi: 10.3389/fimmu.2024.1503853 (PMC11811080; doi:10.3389/fimmu.2024.1503853)
Supplement: Supplementary file 1 [file DataSheet1.docx]

**Supplementary Figures**


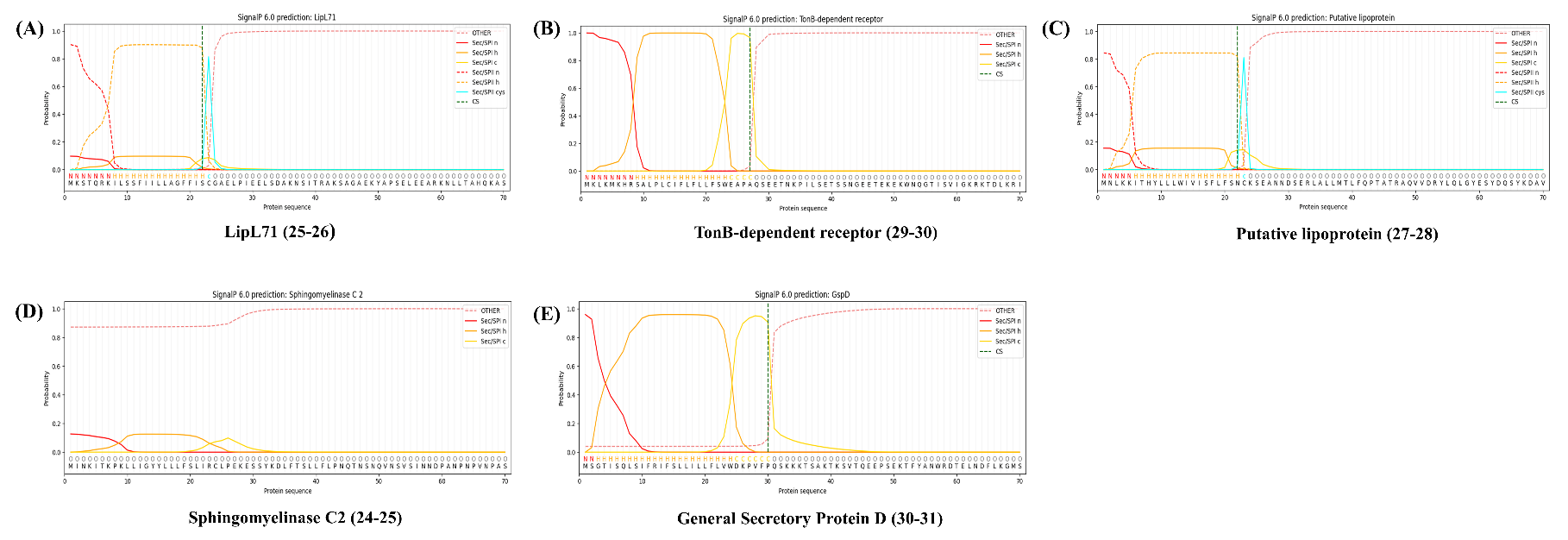


**Supplementary Figure S1.** Predicted signal peptides of the targeted proteins, determined using the SignalP 6.0 server. (A) LipL71. (B) TBDR. (C) irpA. (D) Sph2. (E) GspD. The x-axis represents the sequence number, and the y-axis represents the signal peptide probability score.


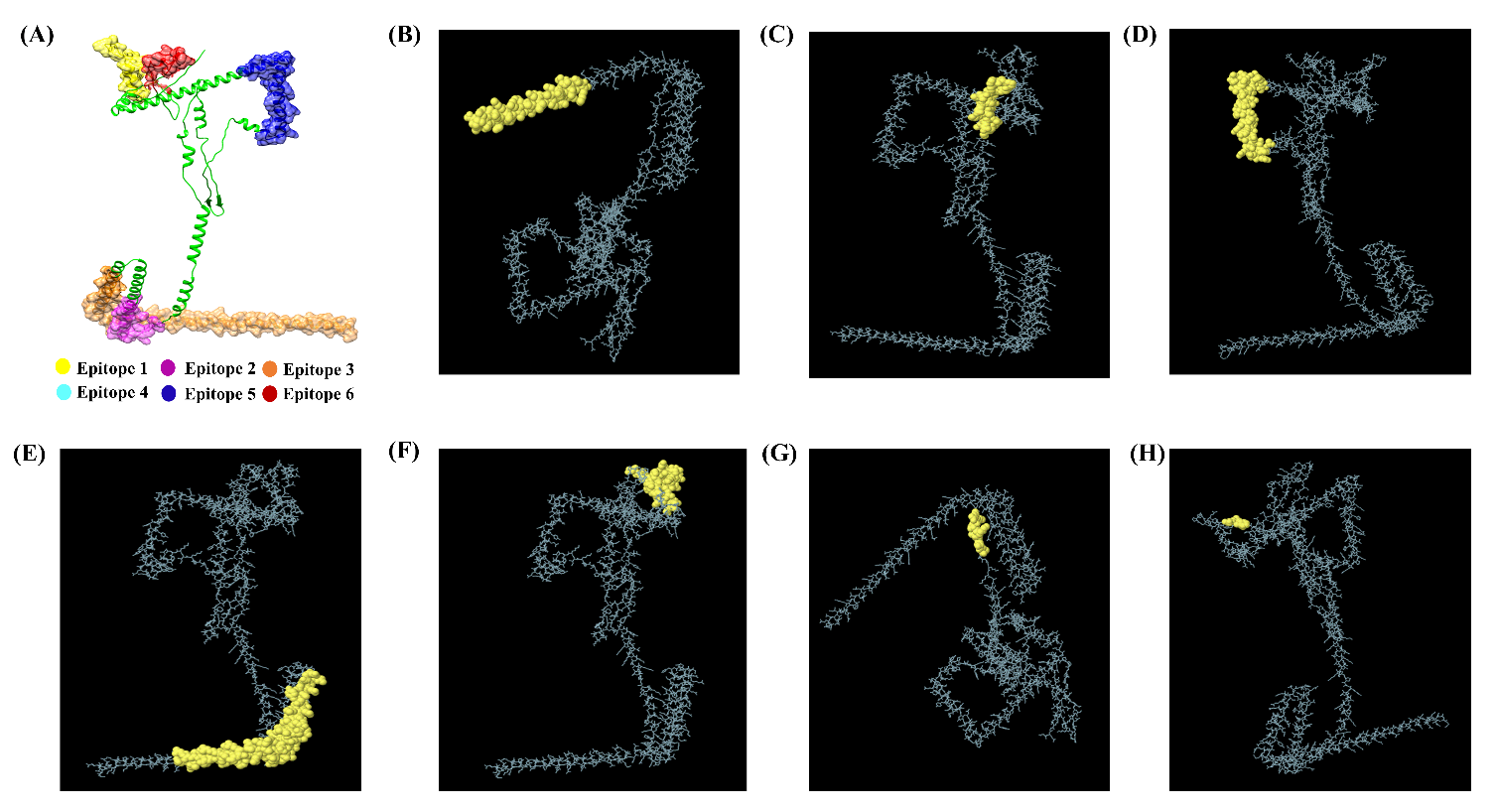


**Supplementary Figure S2.** Continuous and discontinuous antibody epitopes were predicted using the ElliPro tool (A) The ElliPro tool was used to identify six linear (continuous) antibody epitopes on the vaccine’s 3D structure. (B–H) The ElliPro tool predicted seven conformational (discontinuous) antibody epitopes highlighted in yellow in the 3D structure.


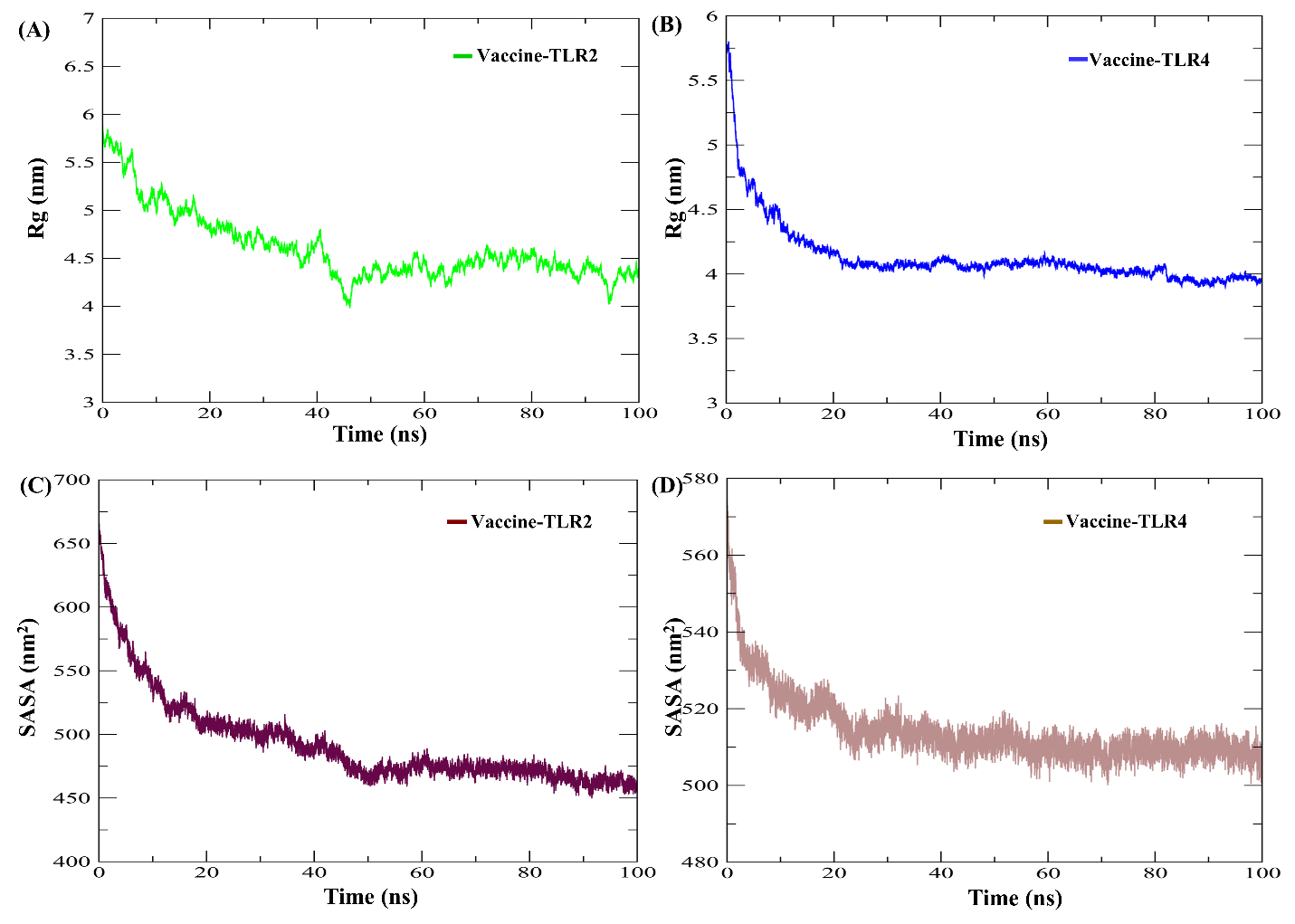


**Supplementary Figure S3.** Radius of Gyration (Rg) and solvent-accessible surface area analysis (SASA) of vaccine-receptor complexes (A) Rg plot for the vaccine-TLR2 complex (green). (B) Rg plot of the vaccine-TLR4 complex (blue). (C) SASA plot of the vaccine-TLR2 complex (dark red). (D) SASA plot of the vaccine-TLR4 complex (brown).


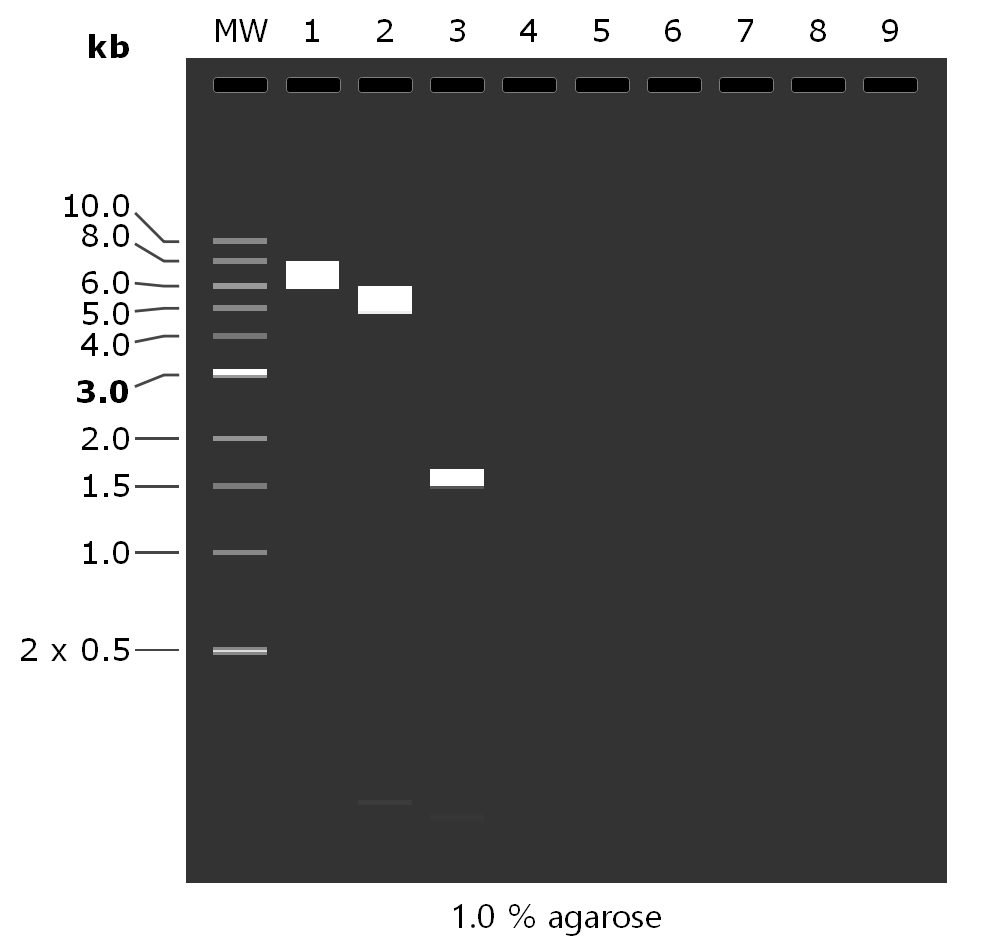


**Supplementary Figure S4.** The 1% agarose gel electrophoresis simulation, where MW, lanes 1, 2, and 3 represent 1 kb of DNA ladder, vaccine (6,874 bp), pET-28a (+) (5,369 bp), and insert (1,551 bp), respectively.
