# Supplementary material for: Designing a broad-spectrum multi-epitope subunit vaccine against leptospirosis using immunoinformatics and structural approaches
Source: Front Immunol. 2025 Jan 28;15:1503853. doi: 10.3389/fimmu.2024.1503853 (PMC11811080; doi:10.3389/fimmu.2024.1503853)
Supplement: Supplementary file 3 [file Table1.docx]

**Supplementary Table S1**. A compilation of databases, software, and web tools was utilized to design a multi-epitope subunit vaccine targeting *Leptospira interrogans*.

| **Database, software, or web service** | **Description** | **Uniform Resource Locator (URL)** |
| --- | --- | --- |
| UniProt | Resource of protein sequence and functional information | <https://www.uniprot.org/> |
| VaxiJen v2.0 | Prediction of antigenicity | <https://www.ddg-pharmfac.net/vaxijen/VaxiJen/VaxiJen.html> |
| PSORTb v3.0.3 | Prediction of sub-cellular localization | <https://www.psort.org/psortb/> |
| CELLO v.2.5 | Prediction of sub-cellular localization | <https://cello.life.nctu.edu.tw/> |
| AllergenFP v.1.0 | Prediction of allergenicity | <https://ddg-pharmfac.net/AllergenFP/> |
| BLASTp | Compares a protein query to a protein database | <https://blast.ncbi.nlm.nih.gov/>  [Blast.cgi?PROGRAM=blastp&PAGE_TYPE=](https://blast.ncbi.nlm.nih.gov/)  [BlastSearch&LINK_LOC=blasthome](https://blast.ncbi.nlm.nih.gov/) |
| SignalP 5.0 | Prediction of Signal peptide and cleavage sites in gram+, gram- bacteria. | <https://services.healthtech.dtu.dk/services/SignalP-5.0/> |
| ABCpred | Prediction of B-cell epitope | <http://crdd.osdd.net/raghava/abcpred/> |
| IEDB MHC II | Prediction of helper T-lymphocyte epitope | <https://tools.iedb.org/mhcii/> |
| IFNepitope | Predicting and designing  interferon-gamma inducing epitopes | <http://crdd.osdd.net/raghava/ifnepitope/predict.php> |
| IL4Pred | Prediction of IL4 inducing peptides | <https://webs.iiitd.edu.in/raghava/il4pred/predict.php> |
| NetCTL 1.2 | Prediction of cytotoxic T-lymphocyte epitope | <https://services.healthtech.dtu.dk/services/NetCTL-1.2/> |
| ToxinPred | Prediction of toxicity | <https://webs.iiitd.edu.in/raghava/toxinpred/> |
| IEDB population coverage | Population coverage prediction | <http://tools.iedb.org/population/> |
| CLC Sequence Viewer 8 | A software package supporting your daily bioinformatics work | <https://resources.qiagenbioinformatics.com/manuals/clcsequenceviewer/current/index.php?manual=Introduction_CLC_Sequence_Viewer.html> |
| WebLogo | A web-based application designed to make the generation of sequence logos | <https://weblogo.berkeley.edu/logo.cgi> |
| ProtParam | Physicochemical properties | <https://web.expasy.org/protparam/> |
| ANTIGENpro | Prediction of antigenicity | <https://scratch.proteomics.ics.uci.edu/> |
| SOLPro | Solubility prediction | <https://scratch.proteomics.ics.uci.edu/> |
| AllerTOP v.2.0 | Prediction of allergenicity | <https://www.ddg-pharmfac.net/AllerTOP/> |
| PSIPRED v4.0 | Secondary structure prediction | <http://bioinf.cs.ucl.ac.uk/psipred/> |
| GOR IV | Secondary structure prediction | <https://npsa-prabi.ibcp.fr/cgi-bin/npsa_automat.pl?page=/NPSA/npsa_gor4.html> |
| trRosetta | Protein structure and function Prediction | <http://raptorx6.uchicago.edu/> |
| GalaxyRefine | Protein structure refinement | <https://galaxy.seoklab.org/cgi-bin/submit.cgi?type=REFINE> |
| ProSA-web | Protein structure  validation | <https://prosa.services.came.sbg.ac.at/prosa.php> |
| PROCHECK | Protein structure  validation | <https://saves.mbi.ucla.edu/> |
| Chimera 1.17.1 | Interactive visualization and analysis of molecular structure | <https://www.rbvi.ucsf.edu/chimera/> |
| IEDB ElliPro | Antibody Epitope Prediction | <http://tools.iedb.org/ellipro/> |
| ClusPro 2.0 | Docking study | <https://cluspro.bu.edu/login.php> |
| LigPlot+ v.2.2.5 | Structural interaction analysis | <https://www.ebi.ac.uk/thornton-srv/software/LigPlus/> |
| GROMACS | A molecular dynamic package mainly designed for simulations of proteins, lipids, and nucleic acids | <https://www.gromacs.org/> |
| HawkDock | A tool for MM-GBSA calculation | <https://cadd.zju.edu.cn/hawkdock/> |
| C-ImmSim | Immune simulation | <https://kraken.iac.rm.cnr.it/C-IMMSIM/index.php> |
| VectorBuilder | Codon optimization | <https://en.vectorbuilder.com/tool/codon-optimization.html> |
| SnapGene | *In-silico* simulation | <https://www.snapgene.com/> |
