# Supplementary material for: Designing a broad-spectrum multi-epitope subunit vaccine against leptospirosis using immunoinformatics and structural approaches
Source: Front Immunol. 2025 Jan 28;15:1503853. doi: 10.3389/fimmu.2024.1503853 (PMC11811080; doi:10.3389/fimmu.2024.1503853)
Supplement: Supplementary file 4 [file Table2.docx]

**Supplementary Table S2.** Five antigenic proteins were selected for vaccine design based on significance.

| **Si. No.** | **Protein name** | **Uniport Id** | **Protein**  **length (A.A)** | **Sub-cellular localization** | **AllergenFP**  **Prediction** | **Antigenicity** |
| --- | --- | --- | --- | --- | --- | --- |
| **1** | LipL71 | Q8F1N5 | 555 | Outer Membrane | Non-allergen | 0.7805 |
| **2** | TBDR | Q8F0M4 | 825 | Outer Membrane | Non-allergen | 0.5546 |
| **3** | irpA | Q8F0M3 | 440 | Outer Membrane | Non-allergen | 0.6619 |
| **4** | Sph2 | P59116 | 623 | Outer Membrane/ Extracellular | Non-allergen | 0.481 |
| **5.** | GspD | Q72S17 | 596 | Outer Membrane | Non-allergen | 0.4736 |
