# Supplementary material for: Designing a broad-spectrum multi-epitope subunit vaccine against leptospirosis using immunoinformatics and structural approaches
Source: Front Immunol. 2025 Jan 28;15:1503853. doi: 10.3389/fimmu.2024.1503853 (PMC11811080; doi:10.3389/fimmu.2024.1503853)
Supplement: Supplementary file 5 [file Table3.docx]

**Supplementary Table S3.** Selected HLA allele reference set of MHC-II using the IEDB server.

| **Human HLA-DR Allele (MHC-II)** |
| --- |
| HLA-DRB1*01:01 |
| HLA-DRB1*03:01 |
| HLA-DRB1*04:01 |
| HLA-DRB1*04:05 |
| HLA-DRB1*07:01 |
| HLA-DRB1*08:02 |
| HLA-DRB1*09:01 |
| HLA-DRB1*11:01 |
| HLA-DRB1*12:01 |
| HLA-DRB1*13:02 |
| HLA-DRB1*15:01 |
| HLA-DRB3*01:01 |
| HLA-DRB3*02:02 |
| HLA-DRB4*01:01 |
| HLA-DRB5*01:01 |
| HLA-DQA1*05:01/DQB1*02:01 |
| HLA-DQA1*05:01/DQB1*03:01 |
| HLA-DQA1*03:01/DQB1*03:02 |
| HLA-DQA1*04:01/DQB1*04:02 |
| HLA-DQA1*01:01/DQB1*05:01 |
| HLA-DQA1*01:02/DQB1*06:02 |
| HLA-DPA1*02:01/DPB1*01:01 |
| HLA-DPA1*01:03/DPB1*02:01 |
| HLA-DPA1*01:03/DPB1*04:01 |
| HLA-DPA1*03:01/DPB1*04:02 |
| HLA-DPA1*02:01/DPB1*05:01 |
| HLA-DPA1*02:01/DPB1*14:01 |
