# Supplementary material for: Designing a broad-spectrum multi-epitope subunit vaccine against leptospirosis using immunoinformatics and structural approaches
Source: Front Immunol. 2025 Jan 28;15:1503853. doi: 10.3389/fimmu.2024.1503853 (PMC11811080; doi:10.3389/fimmu.2024.1503853)
Supplement: Supplementary file 6 [file Table4.docx]

**Supplementary Table S4.** Global population coverage analysis of selected HTL and CTL epitopes.

| **Country** | **Coverage** |
| --- | --- |
| Central Africa | 70.78 |
| East Africa | 71.81 |
| East Asia | 94.72 |
| Europe | 98.64 |
| North Africa | 84.13 |
| North America | 96.17 |
| Northeast Asia | 86.33 |
| Oceania | 92.73 |
| South Africa | 79.84 |
| South America | 83.75 |
| South Asia | 83.69 |
| Southeast Asia | 87.8 |
| Southwest Asia | 82.09 |
| West Africa | 81.76 |
| West Indies | 94.43 |
| World | 95.7 |
