# Supplementary material for: Designing a broad-spectrum multi-epitope subunit vaccine against leptospirosis using immunoinformatics and structural approaches
Source: Front Immunol. 2025 Jan 28;15:1503853. doi: 10.3389/fimmu.2024.1503853 (PMC11811080; doi:10.3389/fimmu.2024.1503853)
Supplement: Supplementary file 7 [file Table5.docx]

**Supplementary Table S5.** Linear or continuous antibody epitopes predicted by ElliPro.

| **Si. No.** | **Start** | **End** | **Peptide** | **Residues** | **Score** |
| --- | --- | --- | --- | --- | --- |
| **1** | 1 | 104 | MAENSNIDDIKAPLLAALGAADLALATVNEL  ITNLRERAEETRTDTRSRV  EESRARLTKLQEDLPEQLTELREKFTAEELRKAAEGYLEAATSRYNELVERGEA | 104 | 0.798 |
| **2** | 414 | 444 | PGPGAEENLKAAEESRVAAGPGPGRSYRFVG | 31 | 0.769 |
| **3** | 308 | 360 | GEEENPENLAAYKVYSAYTERAAYHEVNNTKSLAAYVTSTGPGLKAAYAQMTY | 53 | 0.729 |
| **4** | 462 | 484 | FAANNNPTAADGPGPGGKKFHVI | 23 | 0.71 |
| **5** | 135 | 164 | EALGTVASQTRAVGERAAKLVGIELPKKAA | 30 | 0.639 |
| **6** | 501 | 512 | DNQEAEISVGQD | 12 | 0.576 |
