# Supplementary material for: Designing a broad-spectrum multi-epitope subunit vaccine against leptospirosis using immunoinformatics and structural approaches
Source: Front Immunol. 2025 Jan 28;15:1503853. doi: 10.3389/fimmu.2024.1503853 (PMC11811080; doi:10.3389/fimmu.2024.1503853)
Supplement: Supplementary file 8 [file Table6.docx]

**Table S6.** Predicted conformational or discontinuous antibody epitopes using ElliPro.

| **Si. No.** | **Discontinuous epitope no.** | **No. of residues** | **Score** |
| --- | --- | --- | --- |
| **1** | Epitope 1 | 38 | 0.939 |
| **2** | Epitope 2 | 28 | 0.782 |
| **3** | Epitope 3 | 53 | 0.789 |
| **4** | Epitope 4 | 86 | 0.71 |
| **5** | Epitope 5 | 37 | 0.654 |
| **6** | Epitope 6 | 7 | 0.564 |
| **7** | Epitope 7 | 3 | 0.564 |
